# Acute ischemia induces spatially and transcriptionally distinct microglial subclusters

**DOI:** 10.1186/s13073-023-01257-5

**Published:** 2023-12-11

**Authors:** Huiya Li, Pinyi Liu, Bing Zhang, Zengqiang Yuan, Mengdi Guo, Xinxin Zou, Yi Qian, Shiji Deng, Liwen Zhu, Xiang Cao, Tao Tao, Shengnan Xia, Xinyu Bao, Yun Xu

**Affiliations:** 1 Department of Neurology, Drum Tower Hospital, Medical School and The State Key Laboratory of Pharmaceutical Biotechnology, Institute of Translational Medicine for Brain Critical Diseases, Nanjing University, Nanjing, 210008 China; 2 Department of Radiology, The Affiliated Drum Tower Hospital of Nanjing University Medical School, Nanjing, 210008 China; 3 The Brain Science Centre, Beijing Institute of Basic Medical Sciences, Beijing, 100850 China; 4 Centre of Alzheimer’s Disease, Beijing Institute for Brain Disorders, Beijing, 100069 China; 5 Jiangsu Key Laboratory for Molecular Medicine, Medical School of Nanjing University, Nanjing, 210008 China; 6 Jiangsu Provincial Key Discipline of Neurology, Nanjing, 210008 China; 7 Nanjing Neurology Medical Centre, Nanjing, 210008 China

**Keywords:** Ischemic stroke, Microglia, scRNA-seq, Spatial transcriptomics, BACH1, Glucocorticoids

## Abstract

**Background:**

Damage in the ischemic core and penumbra after stroke affects patient prognosis. Microglia immediately respond to ischemic insult and initiate immune inflammation, playing an important role in the cellular injury after stroke. However, the microglial heterogeneity and the mechanisms involved remain unclear.

**Methods:**

We first performed single-cell RNA-sequencing (scRNA-seq) and spatial transcriptomics (ST) on middle cerebral artery occlusion (MCAO) mice from three time points to determine stroke-associated microglial subclusters and their spatial distributions. Furthermore, the expression of microglial subcluster-specific marker genes and the localization of different microglial subclusters were verified on MCAO mice through RNAscope and immunofluorescence. Gene set variation analysis (GSVA) was performed to reveal functional characteristics of microglia sub-clusters. Additionally, ingenuity pathway analysis (IPA) was used to explore upstream regulators of microglial subclusters, which was confirmed by immunofluorescence, RT-qPCR, shRNA-mediated knockdown, and targeted metabolomics. Finally, the infarct size, neurological deficits, and neuronal apoptosis were evaluated in MCAO mice after manipulation of specific microglial subcluster.

**Results:**

We discovered stroke-associated microglial subclusters in the brains of MCAO mice. We also identified novel marker genes of these microglial subclusters and defined these cells as ischemic core-associated (ICAM) and ischemic penumbra-associated (IPAM) microglia, according to their spatial distribution. ICAM, induced by damage-associated molecular patterns, are probably fueled by glycolysis, and exhibit increased pro-inflammatory cytokines and chemokines production. BACH1 is a key transcription factor driving ICAM generation. In contrast, glucocorticoids, which are enriched in the penumbra, likely trigger IPAM formation, which are presumably powered by the citrate cycle and oxidative phosphorylation and are characterized by moderate pro-inflammatory responses, inflammation-alleviating metabolic features, and myelinotrophic properties.

**Conclusions:**

ICAM could induce excessive neuroinflammation, aggravating brain injury, whereas IPAM probably exhibit neuroprotective features, which could be essential for the homeostasis and survival of cells in the penumbra. Our findings provide a biological basis for targeting specific microglial subclusters as a potential therapeutic strategy for ischemic stroke.

**Supplementary Information:**

The online version contains supplementary material available at 10.1186/s13073-023-01257-5.

## Background

The high mortality and disability rates associated with ischemic stroke are closely related to the extent of neuronal necrosis occurring after cerebral ischemia, where neurons in the ischemic core gradually die (infarct) due to persistent ischemia or ischemic–reperfusion injury. The periphery of the ischemic core, called the penumbra, shows no apparent neuronal death and is salvageable if blood flow is restored. However, in some cases, the penumbra may convert to an infarct. Microglia-mediated immune inflammation plays an important role in this process [1, 2].

Microglia are immune cells resident in the central nervous system, which maintain the brain in a normal physiological and immune-privileged state [3]. Microglia rapidly respond to ischemia and initiate an early immune response. Pro-inflammatory cytokines (e.g., TNF-α) derived from reactive microglia can directly promote neuronal death and blood–brain barrier injury [4]. However, microglia also secrete complement components to tag stressed neurons and engulf these otherwise salvageable neurons in the penumbra [5]. Previous studies have applied the concept of M1/M2 polarization of macrophages to microglia because microglia are the brain’s macrophages [6, 7]. However, arguably, this dichotomy is simply based on in vitro culture conditions, and microglial responses must be determined by in vivo proteomic or transcriptomic analyses in different disease models [8, 9].

To identify stroke-associated microglial subclusters and their marker genes, functions, metabolic states, and their regulatory microenvironmental cues, we, for the first time, combined single-cell RNA sequencing (scRNA-seq) with spatial transcriptomics (ST) to investigate the transcriptional changes of microglia in the brain in a mouse model of ischemic stroke.

## Methods

### *Cell culture and *in vitro* experiments*

#### Primary microglia

Primary glial cells were prepared from 1-day-old C57BL/6 J mice, as previously described [10]. Primary microglia were cultured in Dulbecco’s modified Eagle’s medium (DMEM; Invitrogen, Carlsbad, CA, USA), 10% fetal bovine serum (FBS; HyClone, Logan, UT, USA), and 1% penicillin/streptomycin (100 U/mL; ThermoFisher Scientific, Waltham, MA, USA), which was changed at 36–48 h and 6–7 days, respectively. Primary microglia were cultured in a humidified culture chamber under 5% CO_2_ at 37℃. On day 10, 75-cm^2^ flasks were agitated to separate primary microglia and astrocytes. After cell plating, primary microglia were treated with LPS (100 ng/mL, 3 h), repeatedly freeze-thawing medium of HT-22 cells (3–5 cycles, 7 h), dexamethasone (5 nM, 24 h), or corticosterone (1 μM, 24 h). Cell samples were subsequently collected for further experiments.

#### Oxygen–glucose deprivation in oligodendrocytes

Primary glial cells were obtained from 1-year-old C57BL/6 J mice, as previously described [10]. For oligodendrocyte lineage cell culture, plates were first coated with 0.1 mg/mL poly-D-lysine (Sigma–Aldrich, St Louis, MO, USA) for 5 h, followed by three rinses with sterile ddH_2_O. After drying, the cells were plated according to different requirements. In the first 6 days, the culture medium for primary oligodendrocyte progenitor cells (OPCs) contained 97% DMEM/F12 (Invitrogen), 2% B27 (Thermo Fisher Scientific), 1% penicillin/streptomycin (Thermo Fisher Scientific), 30 ng/mL PDGF (GenScript, China), and 10 ng/mL FGF (GenScript, China). Differentiation was induced on the sixth day using medium supplemented with 20 ng/mL CNTF (GenScript, China) and 50 ng/mL T3 (R&D systems, USA). During oligodendrocyte lineage cell culture, the medium was changed every 2 days.

Oxygen–glucose deprivation experiments were performed on the sixth day after inducing oligodendrocyte differentiation. The anaerobic chamber was pre-sterilized under ultraviolet radiation for 30 min. After exchanging the glucose-containing medium for glucose-free medium, the culture plate was transferred to the chamber. The chamber was balanced with 5% CO_2_ and 95% N_2_ for 10 min to create an anaerobic milieu. The deprivation lasted for 4 h, after which the medium was replaced with either normal or 100 ng/mL recombinant granulin protein containing medium (Sinobiological, Beijing, China). After 18 h reperfusion in a 37℃ chamber, cell samples were collected.

#### Lentivirus-mediated Bach1 knockdown

Bach1-knockdown (Lv-shBach1) and control (Lv-shScr) lentiviruses were obtained from GeneChem (Shanghai, China). The target sequence used was TCAATGCCCAACGGATAATTT. The culture medium for BV2 microglial cells was composed of MEM, 10% FBS, and 1% penicillin/streptomycin. Before lentiviral transfection, a 24-well plate with 5 × 10^4^ cells per well was prepared. The virus and HitransG A (GeneChem, Shanghai, China) were added to the medium (multiplicity of infection = 20). After 8–24 h, the medium was replaced, and 72 h later, fluorescence microscopy and quantitative polymerase chain reaction (PCR) were used to confirm transfection and knockdown effects. If necessary, puromycin (2 μg/mL) was used to improve transfection effects.

#### Animal and experimental model

All animal husbandry and experiments were carried out in compliance with the guidelines and approved by the Animal Care and Use Committee of Nanjing University. Male C57BL/6 J (B6) mice (8-week-old, 20–25 g) were obtained from the Model Animal Research Centre of Nanjing University. Mice were housed in a specialized animal room with a 12-h light/dark cycle and fed standard chow diets.

MCAO surgery was performed as previously described, and all efforts were made to minimize the animals suffering [11]. Mice were first anaesthetized with 0.25% avertin, then fixed on a temperature-controlled heating pad to maintain the body temperature at 37 ± 0.5 ℃. Briefly, the left middle cerebral artery was occluded by inserting a heat-rounded 6/0 nylon suture. Occlusion lasted for 60 min. Laser Doppler flowmetry (Perimed Corp., Stockholm, Sweden) was used to confirm the decrease of cerebral blood flow to 30% of baseline. After 60 min, the suture was withdrawn for brain reperfusion. Mice were then placed on a heating pad to recover. The sham group underwent the same anesthesia and surgical procedures, without suture insertion. MCAO mice were randomly divided into three groups (corticosterone-treated group; RU486-treated group; vehicle-treated group) and were administered corticosterone (10 mg/kg/day), RU486 (30 mg/kg/day), or vehicle at perfusion, and at 24 h or 48 h after surgery, respectively, by intraperitoneal injection. Animals were euthanized with carbon dioxide at 3-h, 12-h, 24-h, or 72-h post cerebral ischemia and tissues were carefully harvested for experiments. Several mice died during the acute stage of cerebral ischemia (three mice in the vehicle-treated group and seven mice in the RU486-treated group).

#### Infarct volume measurement

Mice were anesthetized and decapitated on day 1 after MCAO (*n* = 6/group). Isolated brains were cut into 1-mm-thick coronal sections and were incubated with 2% triphenyltetrazolium chloride (Sigma–Aldrich) for 15 min at 37℃. The slices were then photographed and analyzed using ImageJ software (NIH, Bethesda, MD, USA). The infarct volume was calculated by integration of infarct size of six slices in each brain. The formula for infarct volume calculation was as follows: Infarct size = (contralateral area—ipsilateral non-infarct area) / (contralateral area × 2) × 100%.

#### Neurobehavioral test

A modified neurological severity score (mNSS), ranging from 0 to 12, was assessed on days 1 and 3 after MCAO, based on motor and balance tests. A higher score indicated more severe neurological deficits. A Panlab machine (LE902, Bioseb, Pinellas Park, FL, USA) was used to measure grip strength. All neurobehavioral tests were performed by an investigator who was blinded to the group allocation.

#### Single-cell dissociation and preparation

To prepare single-cell suspensions, we rapidly isolated the ipsilateral hemispheres (ischemic hemisphere) of sham and MCAO mice (MCAO-3 h, MCAO-12 h, MCAO-3d). Brain tissues were carefully kept in MACS Tissue Storage Solution (Miltenyi Biotec, 130–100-008, Germany) and rapidly transferred to Oebiotech (Shanghai, China) for subsequent sample processing and data acquisition. The tissues were then washed with sterile phosphate-buffered saline (PBS) and minced into pieces (< 1mm^3^) on ice. Each sample was enzymatically digested with Trypsin (Gibco, 15,090,046), Type 1 Collagenase (Gibco, 17,018,029), Type 2 Collagenase (Gibco, 17,101,015), and Type 4 Collagenase (Gibco, 17,104,019), followed by centrifugation at 300 rcf for 30 s. After centrifugation, the supernatant was removed and the cell pellet was washed with 1 × PBS containing 0.04% BSA (400 μg/mL); 1 mL red blood cell lysis buffer was then used to resuspend cell pellet for red blood cells lysis. Finally, samples were resuspended in PBS containing 0.04% BSA and filtered through Scienceware Flowmi 40-μm cell strainers (VMR). The hemocytometer and trypan blue staining were employed to determine the cell concentration and cell viability of dissociated single cells.

#### Single-cell sequencing

Briefly, single cells were separately encapsulated in oil droplets after loading into a 10 × Genomics Chromium Controller (Pleasanton, CA, USA) to generate gel beads in emulsions. The mRNA in each droplet was released and reverse transcribed to generate cDNA. To construct a single-cell cDNA library, cDNA was amplified using PCR and further purified using DynaBeads MyOne™ Silane Beads (10 × Genomics). Finally, an Agilent 2100 Bioanalyzer (Santa Clara, CA, USA) was used to determine the size and purity of the cDNA libraries. The scRNA-seq data were deposited in the NCBI SRA database (accession number PRJNA912889) [12].

#### ScRNA-seq data analysis

Using the STAR aligner [13], reads were aligned to the genome and transcriptome after cellular barcode demultiplexing. A matrix of cells and genes was subsequently generated using the CellRanger software pipeline (Version 5.0.0; 10 × Genomics). Strict quality control was performed for each sample. Cells with UMI were filtered and processed using the R package Seurat (Version 3.1.1). Additionally, the gene numbers of each cell were required to be within the mean value ± twofold standard deviations, with a mitochondrial content not exceeding 10%. After excluding low-quality cells for downstream analysis, a total of 32,947 cells were retained for further bioinformatic analysis (9361 cells in the sham group, 8186 cells in the MCAO-3 h group, 9873 cells in the MCAO-12 h group, and 5527 cells in the MCAO-3d group).

#### t-SNE dimension reduction

Based on the log-normalized data, mutual nearest neighbors were used to reduce dimensionality after the elimination of batch effects with the R package (version 3.0). The dimension-reduced results were then visualized using a t-distributed stochastic neighbor embedding (t-SNE) plot. To optimize clustering, unsupervised clustering according to gene expression and dispersion using the shared nearest neighbor algorithm was used [14]. We manually annotated each cluster based on enriched gene composition to determine cell-type.

#### Identification of DEGs

Differentially-expressed genes (DEGs) were identified using the FindMarkers function (test, use = MAST) in Seurat [15]. For DEGs between different clusters, a simultaneous likelihood ratio test was used to estimate the mean expression of genes and percentage of expressing cells. Generally, genes with a *P* value ≥ 0.05, and |log2 fold change|≤ 0.58 were filtered out.

#### AddModuleScore

The AddModuleScore function in Seurat was used to define the scores of the genes of interest. Specifically, the AddModuleScore function calculated the average expression levels of each program (cluster) at the single-cell level, subtracted by the aggregated expression of the control feature sets. All analyzed features were binned based on averaged expression, and control features were randomly selected from each bin.

#### Gene set variation analysis

GSVA, a nonparametric, unsupervised method, was used to evaluate whether different pathways were enriched in different cell populations by converting the expression matrix of genes into the expression matrix of gene sets in different cell populations. Based on the pathway activity score of each cell obtained by GSVA, difference tests were performed on the pathway activity score of each cell group and all other cell groups. The top 10 pathways with the highest t-value in each group, as well as *P* < 0.05, were selected for the heatmap protract.

#### Pseudotime analysis and trajectory inference

For pseudotime analysis, we used the differentialGeneTest function of the Monocle2 package to select ordering genes (qval < 0.01) which were likely to be informative in the ordering of cells along the pseudotime trajectory, simulating dynamic alterations during temporal process.

#### Ingenuity pathway analysis

IPA software (Qiagen, Germantown, MD, USA) was used to perform ingenuity pathway analysis of the top 200 marker genes of IPAM/ICAM to determine key upstream regulators of DEGs.

#### SCENIC analysis

SCENIC analysis was run using the motifs database for RcisTarget and GRNboost (SCENIC version 1.1.2.2, corresponding to RcisTarget 1.2.1 and AUCell 1.4.1) with the default parameters [16]. We identified TF-binding motifs over-represented on a gene list using the RcisTarget package. The activity of each group of regulons in each cell-type was scored using the AUCell package.

To evaluate the cell-type specificity of each predicted regulon, we calculated the RSS, based on the Jensen–Shannon divergence (JSD), a measure of the similarity between two probability distributions. Specifically, we calculated the JSD between each vector of binary regulon activity overlaps with the assignment of cells to a specific cell type [17]. The connection specificity index for all regulons was calculated using scFunctions.

#### Processing and analysis of external datasets

External raw data were downloaded from GSE174574 (Sham vs. MCAO-24 h mice) [18], GSE197731 (ipsilateral and contralateral hemispheres from MCAO-24 h and MCAO-48 h mice) [19], and GSE227651 (Sham vs. MCAO-1d, MCAO-3d and MCAO-7d mice) [20]. Datasets processing including quality control and cell type annotation were performed as described previously [21–23]. Genes expressed in ≥ 3 cells and cells with at least 200 genes expression were included in further analysis. The expression of IPAM/ICAM marker genes (Additional file 6: Table S5) was calculated by the AddModuleScore function in Seurat.

#### Spatial transcriptomic sequencing

Spatial transcriptomic sequencing can be divided into two parts: histology and omics. For sample preparation and optimization, brain tissues were rapidly isolated, frozen, and embedded in optimal cutting temperature compound (OCT). The whole brain was cryo-sectioned coronally (Bregma: 0.98 mm to 1.10 mm) and placed on a spatial tissue optimization slide (10 × Genomics, Pleasanton, CA, USA) containing oligonucleotides to capture RNA after hematoxylin and eosin (H&E)-stained imaging. Each slide had four capture areas with one 6.5 × 6.5-mm capture area containing 5000 spatially barcoded spots. A unique barcode sequence was attached to each 55 × 55-μm spot, with the distance between the centers of each spot being 100 μm. Briefly, mRNA was first released from cells through fixation and enzymatic permeabilization. Released mRNA was labelled with the corresponding barcode sequence. According to the spatial barcode information, we were able to identify the location from which the data derived, enabling the visualization of spatial gene expression. Using the captured mRNA as a template, cDNA was subsequently synthesized using reverse transcription reagents and further amplified for Visium spatial gene expression library (10 × Genomics) construction. We used the NovaSeq 6000 platform to sequence the library by paired-end 150-bp sequencing (PE150). Combined with H&E images, SpaceRanger (Version 1.2.0, 10 × Genomics) identified the expression as well as the spatial location information of genes, which were consequently visualized in the Loupe Browser (Version 5.1.0, 10 × Genomics).

The captured area in the chip was displayed using an image-processing algorithm. The reads from each spot were distinguished based on the spatial barcode information. After comprehensive consideration of the total number of spots, the number of paired reads of each spot, the number of detected genes, as well as UMIs, sample quality could be evaluated by STAR.

Sctransform, an advanced standardization method in Seurat, retains the biological heterogeneity of samples while efficiently removing additive technical influences. Thus, we used Sctransform to normalize the data, construct a regularized negative binomial model of gene expression, and detect high-variance features.

The quantitative result of the spatial transcriptome is an M × N-dimensional matrix. Briefly, average expression and dispersion were calculated for each gene. Principal component analysis was performed to reduce the dimensionality of the log-transformed gene-barcode matrices of the top variable genes. Cells were clustered using a graph-based clustering approach and visualized in two dimensions using t-SNE. To approximate the location of different cell types, we used marker genes of different cell types derived from our scRNA-seq data and employed the AddModuleScore function to calculate the score of the different genes in each spot. The ST data were deposited in the NCBI SRA database (accession number PRJNA912889) [24].

#### RNAscope

For RNAscope analysis, whole brains were collected from MCAO and sham mice. Perfused brains were fixed overnight in 4% paraformaldehyde (PFA), dehydrated in 15% sucrose for 24 h, then in 30% sucrose for another 24 h. Frozen mouse brains were coronally sectioned into 20-μm-thick slices and each section was placed at the center of SuperFrost Plus Slides (Thermo Fisher Scientific, 12–550-15). Prepared brain slices were stored at − 80℃ for further experiments, performed within 3 months.

The RNAscope Multichannel Second Generation Fluorescence kit (ACD, Newark, CA, USA) and RNA–protein Co-Detection Auxiliary kit (ACD) were used to perform RNA in situ hybridization, in accordance with the manufacturer’s instructions. All the agents, equipment, and custom probes were purchased from ACD. Prepared slides were baked at 60℃ for at least 30 min and rinsed with sterile PBS for 5 min to remove OCT. Pre-chilled 4% PFA was used to post-fix the sections for 30 min. Sections were subsequently dehydrated with a gradient of ethanol concentrations (50, 70, 100%) and were then repeatedly dehydrated in 100% anhydrous ethanol. After 5 min of air-drying at room temperature (RT), tissues were incubated with RNAscope hydrogen peroxide for 10 min to block endogenous peroxidases and were then washed twice with distilled water. Next, 0.1% Tween-20 (20 min) was used for brain slice permeabilization. An Immedge™ hydrophobic pen (Vector Laboratories, Burlingame, CA, USA) was used to draw the hydrophobic circle, and sections were blocked with 2% BSA for 1 h. The sections were incubated with the primary antibody (anti-Tmem119, 400 011; Synaptic Systems, Göttingen, Germany) diluted 1:100 in co-detection antibody diluent overnight at 4℃. Then, a HybEZ™ II hybridization furnace (ACD) was preheated to 40℃ for at least 1 h. After rewarming for 30 min at RT, the slides were fixed with fresh pre-cooled 4% PFA for 30 min. The sections were then washed, loaded into the RNAscope EZ-Batch Slide Holder (ACD), and treated with protease PLUS (ACD) for 25 min at 40℃. Probe hybridization was performed by incubating brain sections with custom-designed probes (RNAscope Probe Mm-Gpr65, RNAscope Probe Mm-Srxn1-C3) for 2 h in a HybEZ™ hybridization furnace. Positive and negative controls (RNAscope 3-plex Positive-control Probe-Mm and RNAscope 3-plex Negative-control Probe) were performed in parallel. For signal amplification, sections were incubated with AMP1 for 30 min, AMP2 for 30 min, and AMP3 for 15 min, according to the manufacturer’s instructions. TSA plus fluorophores (ACD) were then used to mark different channels as green (Opal^TM^520 reagent pack) or red (Opal^TM^570 reagent pack) for the detection of RNAscope probes. A fluorescence-conjugated secondary antibody diluted 1:500 was used to mark the corresponding primary antibody at RT, after three changes of PBS, for 2 min each. Finally, the slides were counterstained with DAPI for 30 s at RT.

Confocal microscopy was performed using a confocal fluorescence microscope (Olympus FV3000, Tokyo, Japan). Z-stacks with 1.0-μm spacing were deconvolved and compressed into images (40 ×). Cells with two or more spots corresponding to RNAscope probes were manually judged as positive and were included for statistical analysis.

#### Targeted metabolomics

MCAO surgery was performed as previously described. Twelve hours after perfusion, mice were decapitated and the ischemic hemisphere immediately isolated and rinsed with PBS. Infarct tissues were visually identified by experience, gently peeled off, collected in clean tubes, and rapidly frozen in liquid nitrogen (LN). The remaining hemispheres were considered as infarct-surrounding tissues and were also frozen in LN. All samples were then stored at –80℃.

After weighing, an aliquot from each sample was used for metabolite extraction. An ACQUITY UPLC-I/CLASS-TQ-S (Waters, Shanghai, China) was applied for mass spectrometry (MS). An ACQUITY UPLC BCH C8 LC column (100 × 2.1 mm, 1.7 μm, Waters) was used for chromatographic separation. For assay development, a SCIEX 6500 QTRAP + triple quadrupole mass spectrometer (Sciex, Framingham, MA, USA) equipped with an IonDrive Turbo V electrospray ionization interface was employed. Prior to UHPLC-MRM-MS/MS analysis, a series of calibration standard solutions for each standard substance were prepared and loaded into the MS, providing standard references and optimizing the multiple reaction monitoring parameters. Nineteen types of steroid hormones were targeted, including testosterone, androstenedione, 17α-hydroxyprogesterone, dehydroisoandrosterone sulfate, cortisol, 11-deoxycortisol, 21-deoxycortisol, corticosterone, 11-deoxycorticosterone, dehydroepiandrosterone, dihydrotestosterone, pregnenolone, estrone, 17α-hydroxypregnenolone, cortisone, progesterone, α-oestradiol, β-oestradiol, oestriol, aldosterone, and epiandrosterone. All MS data acquisition and quantitative analysis were completed using MassLynx Workstation Software (Version 4.1, Waters). By measuring the signal-to-noise ratios, the lowest limits of detection for steroid hormones and the lowest limits of quantification ranged from 0.31 to 610.35 pg/mL and from 0.61 to 1220.70 pg/mL, respectively. The correlation coefficients (R^2^) for all targeted compounds were ≥ 0.9956, indicating a good quantitative relationship between the chromatographic peak area and compound concentration. To assess precision and accuracy, the relative standard deviation (RSD) and recovery of QC samples were calculated after five repeated sample loadings. The recoveries determined were 98.1–109.0% for all analyses, with all RSDs below 6.9% (*n* = 6).

#### Immunofluorescence

After transcardial perfusion with PBS and 4% PFA, mouse brains were extracted and post-fixed in 4% PFA. Brains were dehydrated in 15% and 30% sucrose, successively, and frozen at − 80℃ for storage. OCT-embedded brains were sectioned into 20-μm-thick slices and placed on 25 × 75-mm adhesion microscope slides (Citotest, China). After three washes in PBS for 5 min each, sections were permeabilized with 0.25% Triton-X 100 for 20 min. Next, 2% BSA was used to block the sections for 2 h, at RT. The sections were then incubated with primary antibodies at 4℃ overnight. Primary antibodies were directed against Tmem119 (1:500, Synaptic Systems, Germany, 400 011), Tmem119 (1:100, Abcam, USA, ab209064), LGALS3 (1:500, Biolegend, USA, 125,401), PIK3IP1 (1:50, Santa Cruz Biotechnology, USA, sc-365777), HMGB1 (1:500, Abcam, USA, ab79823), and NEUN (1:500, Abcam, USA, ab104224). The next day, slices were rewarmed for 30 min and washed three times in PBS for 10 min each time. Fluorescence-conjugated secondary antibodies against the corresponding host species (1:500, Invitrogen, USA) were used to immunostain the sections for 2 h, in the dark, at RT. Cell nuclei were counterstained with DAPI (5 g/mL, Bioworld, USA, BD5010) for 10 min. Finally, the slides were imaged using a confocal fluorescence microscope (Olympus FV3000) and analyzed using ImageJ software (NIH).

#### TUNEL staining

A TUNEL apoptosis detection kit (Orange Fluorescence) (Abbkine, China) was used to perform TUNEL staining. Briefly, sections were permeabilized with 0.25% Triton X-100 for 20 min, blocked with 2% BSA for 1 h, incubated with 1 × equilibration buffer for 20 min, and stained with the reaction mixture for 1 h at 37℃ in the dark. After three rinses with PBS, brain sections were stained using a primary antibody against NeuN (1:500, Abcam, USA, ab104224) and the corresponding secondary antibody, as mentioned above.

#### Real-time quantitative PCR

Total RNA was extracted using TRIzol™ reagent (Invitrogen), according to the manufacturer’s protocol. To evaluate RNA concentration and quality, a micro-spectrophotometer (Nano-100, Allsheng, China) was used. Before RNA was reverse transcribed, genomic DNA was removed with a DNA wiper mix. cDNA was prepared using a PrimeScript RT Reagent Kit (Vazyme, China). The 10-μL reaction mix for real-time quantitative PCR (RT-qPCR) consisted of cDNA, primers, RNase-free H2O, and SYBR Green mix (Applied Biosystems, USA). All RT-qPCR assays were performed in duplicate. Gapdh was used as an endogenous housekeeping control for normalization. RT-qPCR was performed using a Step One Plus PCR system (Applied Biosystems, USA). The reaction parameters were pre-incubation (95℃, 30 s), 40 cycles of two-step amplification (95℃ for 5 s and 60℃ for 30 s), and melting (95℃ for 15 s; 60℃ for 60 s; and 95℃ for 15 s).

To evaluate the expression of target genes, delta threshold cycle (ΔCT) values were calculated with reference to the internal control Gapdh. Double delta CT (ΔΔCT) values were calculated in relation to the mean ΔCT of the control samples, indicating the expression levels of the genes of interest. The related primer sequences are listed in Additional file 1: Table S6.

#### Western blotting

Proteins were homogenized in RIPA lysis buffer (Thermo Fisher Scientific, USA) containing protease and phosphatase inhibitors. Samples were centrifuged at 1600 × *g* for 30 min, the supernatant collected, and the concentration quantified using a BCA protein assay kit (Invitrogen, USA). Protein samples were mixed with 5 × loading buffer and heated at 100℃ for 5 min. Twenty micrograms of protein was separated by 10% sodium dodecyl sulfate–polyacrylamide gel electrophoresis and then transferred to polyvinylidene fluoride membranes. Membranes were blocked with 5% skimmed milk for 2 h, at RT, and incubated with primary antibodies, including anti-MAG (Abcam, ab89780, USA) and anti-β-actin (Bioworld, AP0060, USA) at 4℃ overnight. The next day, secondary antibodies against the corresponding host species (1:10,000, Bioworld, USA) were used to incubate the membranes. Finally, the separated proteins were visualized using a chemiluminescence reagent and Gel-Pro System (Tanon Technologies, China). ImageJ software (ImageJ 1.5, NIH) was used to quantify the intensity of bands.

### Statistical analysis

GraphPad Prism (Version 8.0, USA) was used to perform statistical analysis of quantitative data. Data are presented as mean ± SEM. The Mann–Whitney or Student’s *t*-test were used to determine differences between two groups. To evaluate one factor among three or more groups, one-way analysis of variance, followed by Tukey’s post hoc test or the Kruskal–Wallis test, was used. Two-way analysis of variance was used to test the statistical significance of two factors among multiple groups. *P* < 0.05 was considered statistically significant.

## Results

### Identification of cell types

**Fig. 1:**
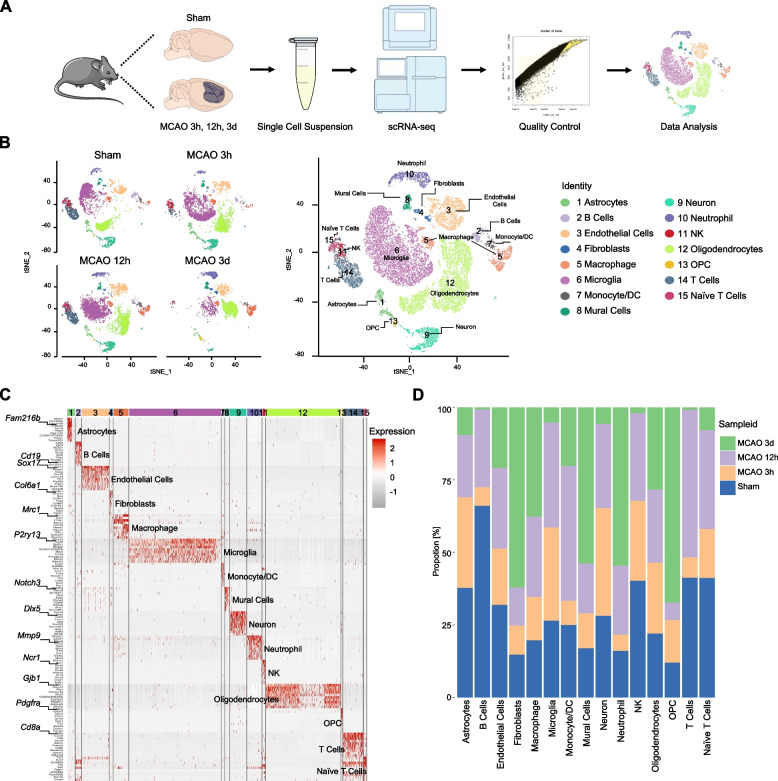
Identification of cell types. **A** Scheme of scRNA-seq. **B** t-SNE plot showing cell clusters in sham and MCAO (3-h, 12-h, and 72-h post-ischemic stroke) mouse brains. Colors highlight 15 transcriptionally distinct clusters. Cell types were manually annotated based on canonical marker gene expression (Additional file 2: Table S2). **C** Heatmap of top 10 marker genes enriched in each cell type based on bimod inspection. Genes of interest are highlighted. Cluster annotations and coloring are consistent across panels. **D** Proportional histogram depicting the proportion alterations of 15 transcriptionally distinct cell types at the acute stage of ischemic stroke (Sham, MCAO-3h, MCAO-12h, and MCAO-72h)

To characterize the changes of the main cell types comprehensively in the ischemic hemisphere following ischemic stroke, we employed droplet-based scRNA-seq (10 × Genomics) on cells isolated from brain samples of mice subjected to transient middle cerebral artery occlusion (MCAO), at different time points post-reperfusion (3-h, 12-h, and 72-h), as well as the sham mouse (Fig. 1A). The 3-h and 12-h time points represent the hyperacute phase of ischemic stroke, while the 72-h time point was selected for transcriptional analysis of acute phase alterations. In total, 39,333 cells with a mean of 1672–8800 unique molecular identifiers (UMIs) per cell were assessed with strict CellRanger (10 × Genomics) quality control (sham: 9361 cells, 3-h: 8186 cells, 12-h: 9873 cells, 72-h: 5527 cells). The average number of genes per cell was 901–2636 among the four groups (Additional file 1: Table S1).

Next, we performed unsupervised clustering and annotated cell types based on canonical gene marker expression (Additional file 2: Table S2). As depicted in the t-distributed stochastic neighbor embedding (t-SNE) plot, 15 distinct cell types, consisting of B cells (563 cells), endothelial cells (2,751 cells), mural cells (448 cells), natural killer (NK) cells (298 cells), monocyte/dendritic cells (DC) (252 cells), oligodendrocyte precursor cells (OPC) (116 cells), T cells (2026 cells), astrocytes (784 cells), fibroblasts (311 cells), macrophages (1545 cells), microglia (9396 cells), neurons (1680 cells), neutrophils (1474 cells), oligodendrocytes (7646 cells), and naive T cells (267 cells) were identified (Fig. 1B). Notably, microglia, oligodendrocytes, and endothelial cells accounted for the majority of the cells. We identified all the marker genes for each cell-type and displayed the top 10 marker genes in a heatmap (Additional file 3: Table S3 and Fig. 1C).

Consistent with previous findings, there was a gradual increase in the number of infiltrating macrophages and neutrophils after ischemic insult, indicating the dysfunction of blood–brain barrier [25]. Besides, the amount of OPC significantly increased at 72-h post-surgery, suggesting the proliferation of OPC after stroke, which showed consistency with previous publications [26]. We also observed a gradual decline in the number of astrocytes after ischemia and a remarkable decrease in microglia at 72-h, which was probably attributed to the acute ischemic damage (Fig. 1D). Collectively, these data provided a basic description of the cellular composition of the ischemic hemisphere.

### Ischemia induces two types of microglial subclusters

**Fig. 2:**
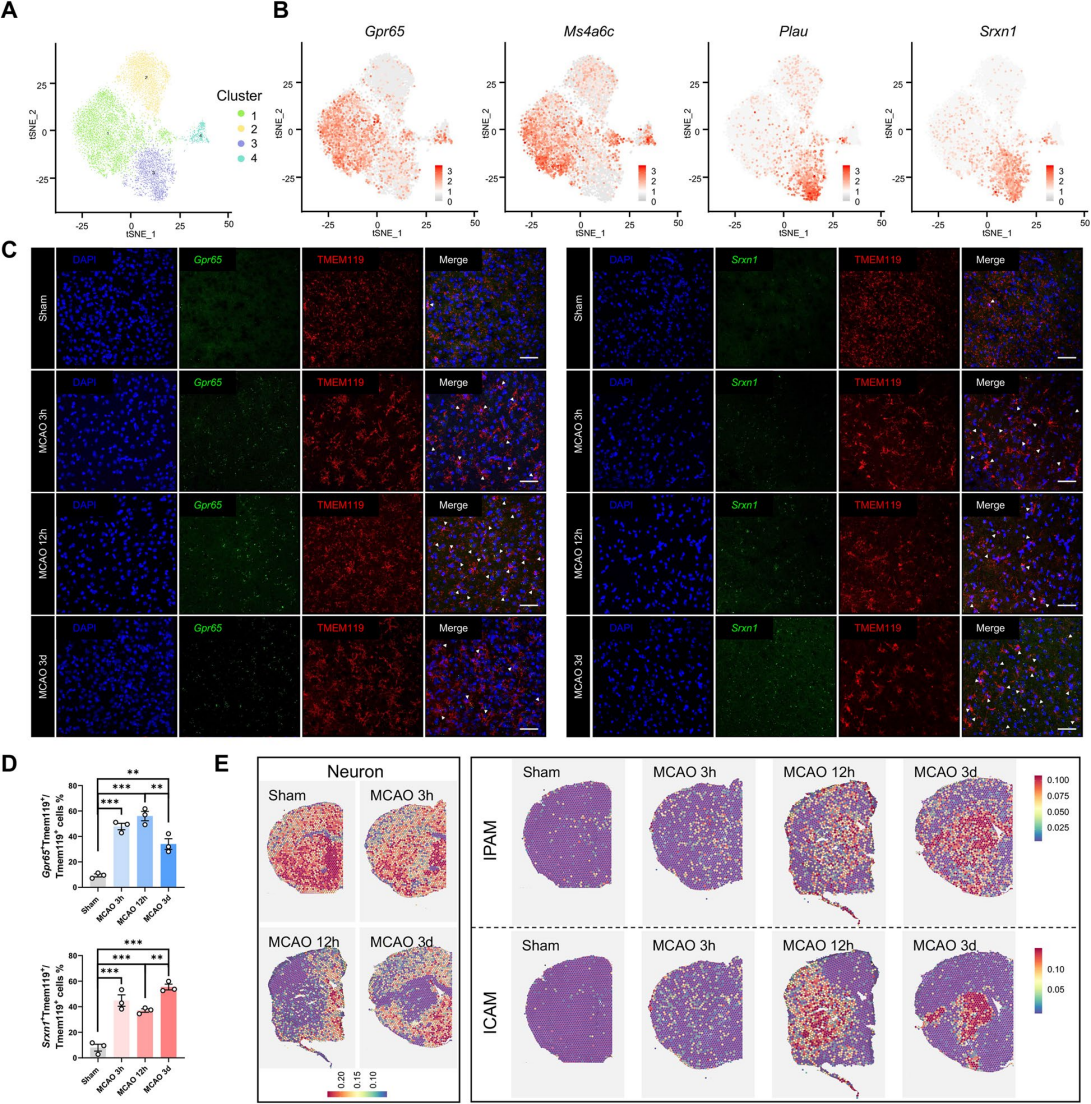
Ischemia induces two microglial subclusters. **A** t-SNE projection of microglia (9396 cells), sub-clustered into four sub-populations (Cluster 1: 4554 cells; Cluster 2: 2362 cells; Cluster 3: 2015 cells; Cluster 4: 465 cells). **B** Feature plots of selected marker genes enriched in Cluster 1 or Cluster 3. **C–D** Representative RNAscope images **(C)** co-stained with *Gpr65*/*Srxn1* and microglial-marker TMEM119 within ischemic penumbra or core in sham and MCAO (3-h, 12-h, 3-day post-ischemic stroke) mice (scale bar: 50 µm; *n* = 3/group). Data are presented as mean ± SEM **(D)**. **P < 0.01, ***P < 0.001, by Student’s *t*-test. **E** 10 × Visium spatial transcriptomics highlighting the presence of Cluster 1 (IPAM) and Cluster 3 (ICAM) in sham (control) and MCAO (3-h, 12-h, 3-day post-ischemic stroke) mouse brain sections, identified by marker genes from scRNA-seq using the AddModuleScore. NeuN was used to define the ischemic lesion

Considering the importance of microglia in the brain and the high quality of microglial transcriptomes, we focused on microglia at the single-cell level (Additional file 5: Fig S1). We identified microglial DEGs at different time points (Additional file 4: Table S4). Consistent with previous findings [27–29], KEGG analysis of microglial DEGs indicated strengthened phagocytosis and inflammation as well as altered metabolism in microglia at the acute stage of ischemic stroke (Additional file 5: Fig S2). Additionally, microglia were categorized into four subclusters for further analysis (Fig. 2A). According to the subcluster proportional diagram, 94% microglia in the sham group was Cluster 2 (Additional file 5: Fig S3A, B). Moreover, the homeostatic genes of microglia (*Tmem119*, *P2ry12*, *P2ry13*, *Csf1r*, *Cx3cr1*, *Hexb*) were highly expressed in Cluster 2 (Additional file 5: Fig S3C), indicating that Cluster 2 probably consisted of homeostatic microglia. Cluster 4 (465 cells) comprised a relatively small number of microglia, which showed little change between sham and MCAO mice; thus, the phenotypic and functional features of Cluster 4 were not further investigated. Clusters 1 (4554 cells) and 3 (2015 cells) accounted for the majority of microglia in the MCAO group (Additional file 5: Fig S3A, B). These findings positioned Clusters 1 and 3 as critical role players in acute ischemic stroke and raised the question of how they orchestrated ischemic attack adaptations.

Differential gene expression analysis was performed to identify subcluster-specific marker genes. Most of the highly expressed genes were also sub-cluster-specific, indicating unique gene expression patterns of microglial subclusters (Fig. 2B, Additional file 5: Fig S3D and Additional file 6: Table S5). Cluster 1 expressed previously unreported marker genes (e.g., *Pik3ip1*, *Cd300lf*, *Gpr65*, *Ms4a6c*, *Cep152*, *Ddit4*, *Zbtb16*, *Abhd15*, *Bmf*, and *Arhgap24*) (Additional file 5: Fig S4). In Cluster 3, expression of *Lgals3*, *Plau*, *Srxn1*, *Ankrd33b*, *Gas2l3*, *Nes*, *Edn1*, *Fam129b*, *Vat1*, and *Fmn1* was upregulated (Additional file 5: Fig S5). The top 10 cluster-specific marker genes could reliably identify microglial subclusters 1 and 3 (Additional file 5: Fig S3E, F). Additionally, disease-associated microglia (DAM) were previously identified as a unique microglial subtype associated with Alzheimer’s disease (AD) [30]. We examined the expression levels of the identified DAM marker genes in our dataset (Additional file 5: Fig S6). Expression of several genes (e.g., *Tyrobp*, *Trem2*, *Apoe*, *Timp2*, and *B2m*) remained unaltered among the four clusters. Expression of several genes (e.g., *Lgal3*, *Fth1*, *Cstb*, *Csf1*, *Cd63*, *Cd9*, *Ccl3*, and *Lilrb4a*) was upregulated in Cluster 3, while their expression remained unchanged in Cluster 1 as compared to homeostatic microglia (Cluster 2), indicating that Cluster 3 may exhibit DAM-like signatures.

To determine whether there were any temporal characteristics in Clusters 1 and 3, we employed RNA–protein co-staining with RNAscope (ACD, Newark, CA, USA), using the Cluster 1-specific marker gene *Gpr65* and Cluster 3-specific marker gene *Srxn1* to label different microglial populations (Fig. 2C). Both Clusters 1 and 3 acted as rapid responders to the ischemic insult after reperfusion (Fig. 2D). To confirm whether Clusters 1 and 3 also existed in other scRNA-seq research data on ischemic stroke, we reanalyzed several existing microglial datasets post-stroke (GSE174574, GSE197731, GSE227651) [18–20]. Consistent with our results, Clusters 1 and 3 could be clearly distinguished in their t-SNE microglia plot (Additional file 5: Fig S7).

To determine whether Clusters 1 and 3 have any spatial relationship with the infarct core, we employed spatial transcriptomics (ST) (10 × Genomics) to study brain slices from the sham and MCAO mice. Although ST hardly reaches single-cell resolution, the approximate location of different cellular clusters in brain sections could still be obtained by calculating the average expression of subcluster-specific marker genes pertaining to scRNA-seq data. To define the ischemic core, we also queried neuronal genes whose downregulation indicated neuronal loss in each spot on the brain slices. Strikingly, Cluster 3 markers occupied the ischemic core, while Cluster 1 markers surrounded the infarct lesion at 12-h post-ischemia (Fig. 2E). Therefore, we defined Cluster 1 as ischemic penumbra-associated microglia (IPAM) and Cluster 3 as ischemic core-associated microglia (ICAM).

To validate these findings, ICAM-specific LGALS3 was used as an ICAM marker, whereas PIK3IP1 was selected as an IPAM marker in an immunofluorescence assay (Additional file 5: Fig S8). ICAM markers were significantly enriched in the ischemic core at 12-h and 72-h. The localization of IPAM markers in the ischemic penumbra at 12-h was also confirmed. Besides, the ICAM-specific marker (*Lgals3*) and IPAM-specific markers (*Pik3ip1*, *Gpr65*, and *Ms4a6c*) were also visualized in the ST (Additional file 5: Fig S9A-D). We also categorized the brain section into two regions (ischemic core and penumbra) based on Fig. 2E. The upregulated genes within the ischemic core were defined as ischemic core genes (ICGs), and the upregulated genes in the ischemic penumbra were named ischemic penumbra genes (IPGs). There were more IPAM markers (199 vs. 158) overlapped with IPGs, and more ICAM markers (397 vs. 297) overlapped with ICGs, further validating the spatial distribution of ICAM and IPAM (Additional file 5: Fig S10A). Thus, our data revealed two spatially and transcriptionally distinct microglial subclusters involved in the response to ischemic stroke.

### ICAM and IPAM exhibit distinct features

**Fig. 3:**
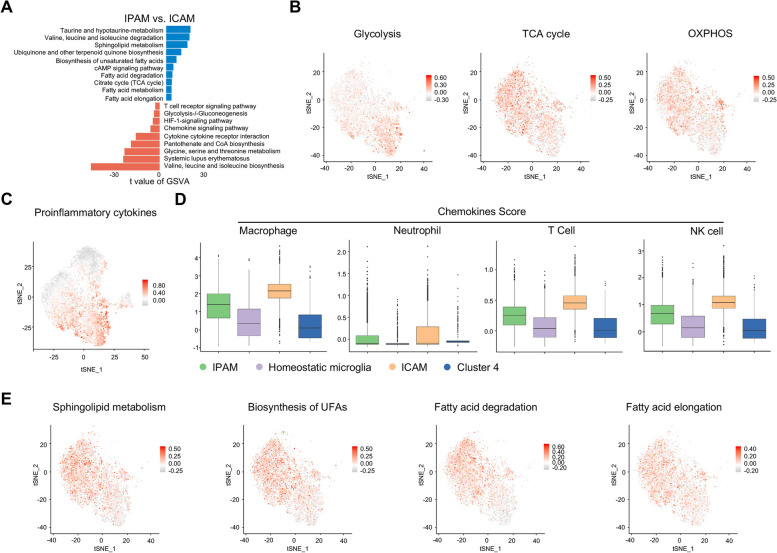
ICAM and IPAM exhibit distinct features. **A** GSVA showing biological processes differentially enriched in IPAM/ICAM. **B** Gene score feature plots in IPAM and ICAM using representative genes involved in glycolysis/TCA cycle/OXPHOS pathways (Additional file 2: Table S6). C Gene score feature plot for pro-inflammatory cytokines (*Il1a*, *Il1b*, *Il6*, *Il18*, *Tnf*, *Hmox1*, *Ptgs2*) in microglia. **D** Boxplots depicting macrophage/neutrophil/T cell/NK-specific chemokine expression (Additional file 2: Table S8) among four microglial subclusters using the AddModuleScore. **E** Gene score feature plots in IPAM and ICAM using canonical sphingolipid metabolism, UFA biosynthesis, fatty acid degradation, and fatty acid elongation marker genes (Additional file 2: Table S6)

For functional assays, we performed gene set variation analysis (GSVA) of IPAM and ICAM (Fig. 3A). We found that ICAM presumably increased their reliance on glycolysis, whereas IPAM probably relied on the tricarboxylic acid (TCA) cycle and oxidative phosphorylation (OXPHOS), which was confirmed by the expression of genes typically associated with the metabolic pathways of interest, as calculated by using the AddModuleScore [31] in Seurat (Fig. 3A, B andAdditional file 2: Table S6). Consistently, “Oxidative phosphorylation” and “Citrate Cycle” were also enriched in IPG, as revealed by KEGG analysis (Additional file 5: Fig S10B). The “broken” TCA cycle in ICAM may also contributed to increased levels of intermediates, as revealed by enhanced pantothenate and CoA biosynthesis in ICAM. Additionally, upregulation of the AMPK and cAMP signaling pathways was observed in IPAM, indicating that IPAM used a completely different energy source (Fig. 3A and Additional file 2: Table S7). Furthermore, metabolic reprogramming of microglia depends on the HIF-1α pathway, which is also highly expressed in ICAM (Fig. 3A and Additional file 2: Table S7).

We further identified increased expression of several pro-inflammatory and inflammation-responsive genes (*Il1a*, *Il1b*, *Il6*, *Il18*, *Tnf*, *Hmox1*, *Ptgs2*) in ICAM as compared to other subclusters (Fig. 3C). However, there was no significant difference in the expression of anti-inflammatory cytokines (*Il4*, *Il10*, *Tgfb1*) among four microglial subclusters (Additional file 5: Fig S11A). Additionally, macrophages and neutrophils began to infiltrate the brain 12-h after MCAO and gathered in the infarct core at 72 h, suggesting that these peripheral immune cells presented a spatial overlap with ICAM (Additional file 1: Fig S11B). As microglia are the main source of various chemokines in ischemic stroke, we examined the expression of a series of chemokines that drive the recruitment of different peripheral immune cells in ICAM and IPAM (Additional file 2: Table S8). Accordingly, the expression of relevant chemokine gene sets in different peripheral immune cells was enriched in ICAM (Fig. 3D). Specifically, *Ccl2*, *Ccl3*, *Ccl4*, *Ccl5*, *Cxcl2*, and *Cxcl16* expression levels were higher in ICAM than in IPAM (Additional file 5: Fig S11C). Furthermore, KEGG analysis consistently showed that pro-inflammatory signaling pathways (“TNF signaling pathway,” “IL-17 signaling pathway,” “MAPK signaling pathway”) were significantly enriched in ICGs (Additional file 1: Fig S10C). These findings indicate excessive pro-inflammatory and chemotactic responses mediated by ICAM, suggesting that ICAM might be detrimental and might accelerate tissue damage during the acute stage of ischemic stroke.

Additionally, compared with ICAM, several metabolic pathways related to amino acids, lipids, and carbohydrates were significantly enriched in IPAM, as shown by GSVA analysis (Fig. 3A and Additional file 2: Table S7). Levels of valine, leucine, and isoleucine, which are branched-chain amino acids (BCAAs), are elevated in ICAM, while BCAA degradation is enhanced in IPAM (Fig. 3E). Furthermore, the metabolism of other amino acids, such as taurine, was enriched in IPAM (Fig. 3A). According to GSVA data, lipid metabolism is also active in IPAM, including sphingolipid metabolism, primary bile acid biosynthesis, biosynthesis of unsaturated fatty acids, and fatty acid degradation and elongation, as confirmed by AddModuleScore (Fig. 3A, 3E, Additional file 2: Table S6 and Table S7). Additionally, metabolic pathways (“BCAAs degradation,” “Biosynthesis of unsaturated fatty acids,” “Fatty acids degradation,” “Fatty acid elongation,” “Sphingolipid metabolism”) enriched in IPAM also showed a penumbral distribution in the ST (Additional file 5: Fig S9E-I).

Since lipid metabolites are essential components of myelin, IPAM may compensate for the oligodendrocytes to supply lipid components. Furthermore, we used CellPhoneDB [32] to analyze ligand–receptor interactions in IPAM–oligodendrocyte interactions (Additional file 5: Fig S11D). Several ligand–receptor pairs have been identified, with GRN–SORT1 being one of the most prominent pairs. To test whether GRN has oligodendrotrophic potential, we treated oligodendrocytes with recombinant mouse GRN (rmGRN) and found that rmGRN significantly promoted MAG expression (Additional file 5: Fig S11E, F).

Taken together, these distinct signatures at least partly shaped microglial responses and provided a new perspective for understanding different activation states of microglial subclusters in response to ischemia stimulation.

### M1/M2 dichotomy fails to classify microglial subclusters after ischemia

**Fig. 4:**
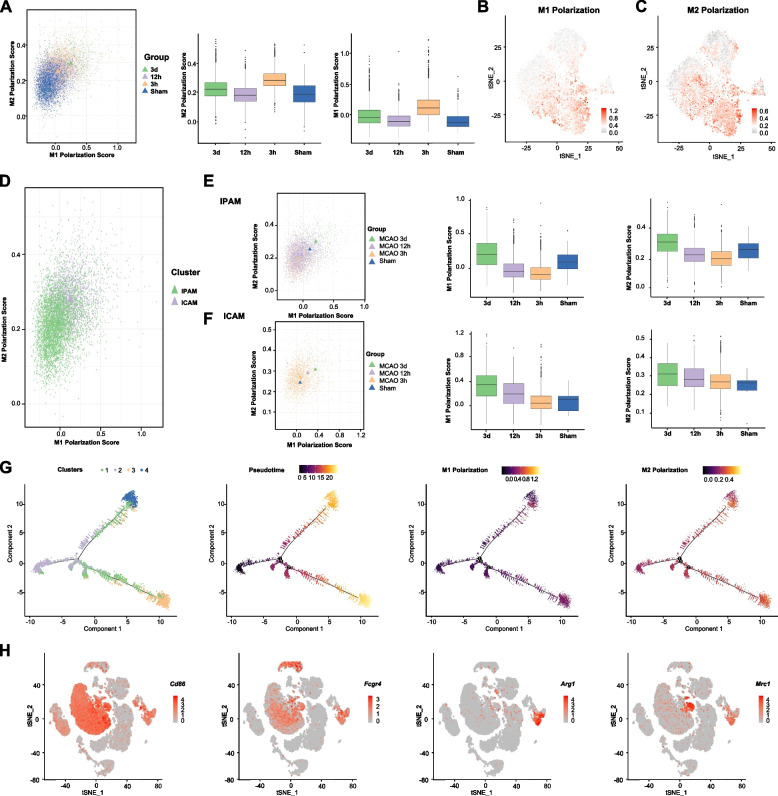
M1/M2 dichotomy fails to classify microglial subclusters after ischemia.** A** Median correlation plot showing M1 polarization score and M2 polarization score of microglia from sham group and MCAO (3-h, 12-h, and 3-day post stroke) group using Corscatter (Additional file 2: Table S9). M1 polarization score and M2 polarization score of microglia from sham group and MCAO (3-h, 12-h, and 3-day post stroke) group were depicted in the box plot. **B** Gene score feature plot using M1-microglial marker. **C** Gene score feature plot using M2-microglial marker genes. **D** Median correlation plot showing M1 polarization score and M2 polarization score of IPAM and ICAM using Corscatter. **E** Median correlation plot showing the alterations of M1 polarization score and M2 polarization score in IPAM among different groups (sham, MCAO-3 h, MCAO-12 h, and MCAO-3d). **F** Median correlation plot showing the differences of M1 polarization score and M2 polarization score in ICAM among different groups (sham, MCAO-3 h, MCAO-12 h, and MCAO-3d). **G** Pseudo-time trajectory diagram of microglia. The expression of M1/ M2 microglial marker genes was depicted in the diagram respectively. **H** t-SNE projections highlighting M1-marker (*Cd86* and *Fcgr4*) and M2-marker (*Arg1* and *Mrc1*)

Historically, based on in vitro stimulations, microglia have been simply classified as pro-inflammatory “M1” and anti-inflammatory “M2”. However, according to our data, the increase of M1 polarization score was always accompanied with escalated M2 polarization score at different time points following ischemia (Fig. 4A). We wondered whether our unsupervised clustering corresponded with this phenotype classification. To test this assumption, we performed M1/M2 polarization score on IPAM and ICAM respectively. However, both M1 and M2 polarization score of ICAM were higher than those of IPAM (Fig. 4B-D). Besides, IPAM and ICAM exhibited the same tendency of M1/M2 polarization alteration over time following ischemic insult (Fig. 4E-F).

Next, we also performed pseudo-time trajectory analysis and observed a distinct diverging trajectory after ischemia. To elucidate whether these two branches were in accordance with M1- and M2-phenotype microglia, we examined the expression of M1/M2 marker genes in different trajectories. There was still no significant difference in the M1/M2 marker genes expression between these two branches (Fig. 4G). Based on numerous published in vivo studies on M1/2 microglia, we noticed that the researchers used Iba-1, which was expressed not only in microglia but also macrophages, as a microglial marker. They also employed CD86 and CD16 (encoded by *Fcgr4*) to label “M1” microglia, Arg1 and CD206 (encoded by *Mrc1*) to label “M2” microglia. However, according to our scRNA-seq data, “M1” microglial markers (*Cd86* and *Fcgr4*) were expressed in both microglia and macrophages, while “M2” microglial markers (*Arg1* and *Mrc1*) were mainly expressed in macrophages, indicating that infiltrating macrophages were probably misidentified as classically defined “M2” microglia (Fig. 4H). Thus, M1/M2 dichotomy might be unable to identify distinct microglial subpopulations and depict their functions after ischemia.

### Key DAMPs and regulons drive ICAM generation

**Fig. 5:**
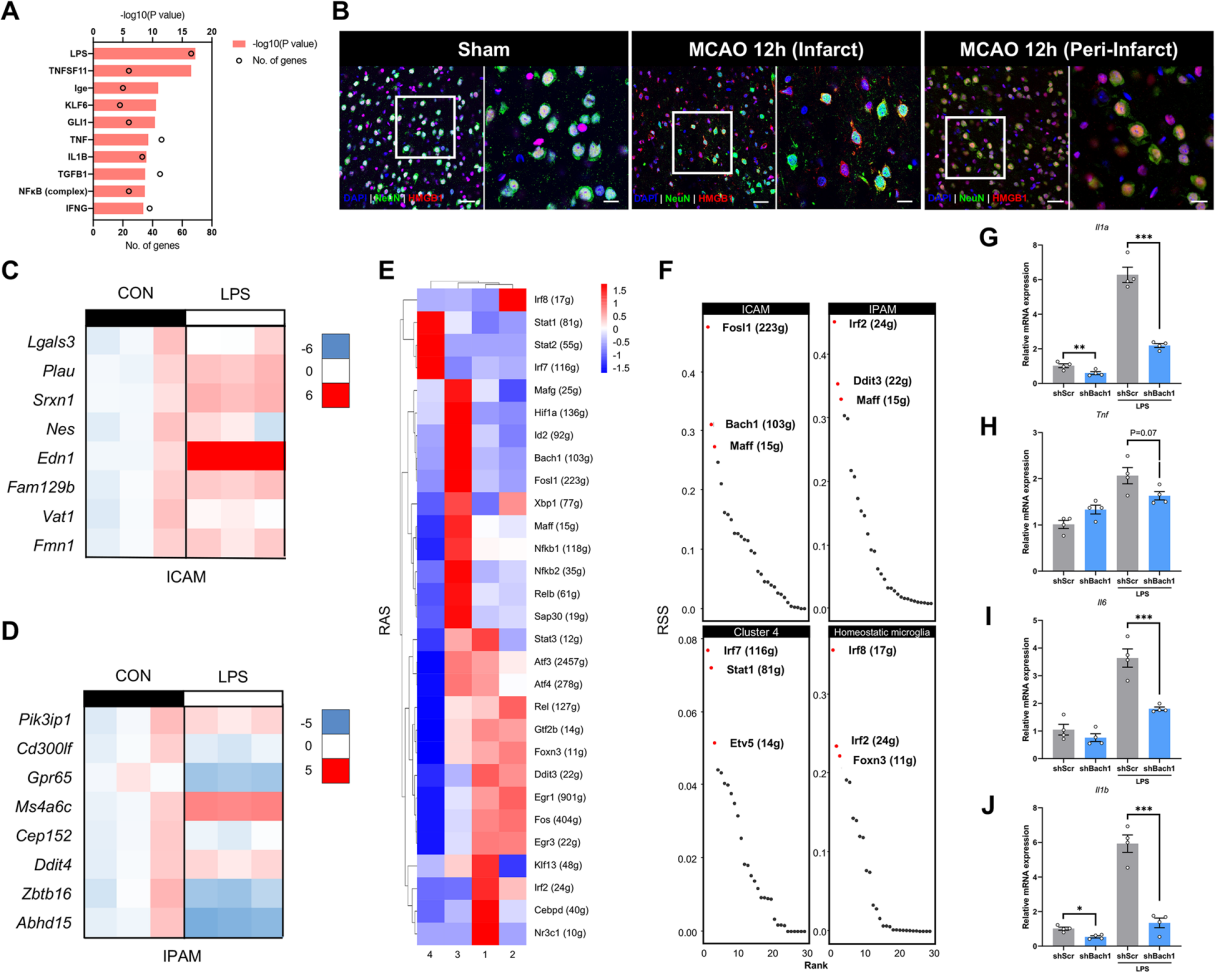
Key DAMPs and regulons drive ICAM generation. **A** Upstream IPA of top 200 differentially expressed genes in ICAM (Fisher’s exact test, Benjamini–Hochberg FDR). **B** Representative confocal immunofluorescence images showing HMGB1 and NEUN expression within the infarct or peri-infarct in sham or MCAO-12 h mouse brains (scale bar: 50 µm). The right panels are magnified (scale bar: 20 µm). **C–D** Heatmap showing ICAM-specific (**C**) or IPAM-specific (**D**) marker genes expression upon LPS stimulation (100 ng/mL, 3 h). *n* = 3/group. **E**–**F** Heatmap (**E**) and ranking plot (**F**) depicting the RAS and RSS of different regulons among four microglial sub-clusters using SCENIC analysis. A transcription factor and its potential targeted genes were regarded as a regulon. RAS represents regulon activity. RSS indicates regulon specificity. **G**–**J** Real-time PCR analysis of the expression of pro-inflammatory factors (*Il1a*, *Tnf*, *Il6*, and *Il1b*) in shScr/shBach1 cell line upon or without LPS stimulation (500 ng/mL, 12 h). *n* = 4 biological independent samples. Data are presented as mean ± SEM. *P < 0.05, **P < 0.01, ***P < 0.001, by Student’s *t*-test

We hypothesized that regional microenvironmental cues might drive the metabolic and functional shifts of microglia towards ICAM and IPAM. By employing Ingenuity System Pathway Analysis (IPA) software (QIAGEN), we found that lipopolysaccharides (LPS) were one of the greatest potential factors driving ICAM generation (Fig. 5A). Since LPS is a canonical ligand that activates TLR4, we investigated the spatial distribution of HMGB1, an endogenous ligand of TLR4 released by damaged cells, after cerebral ischemia. Obvious cytoplasmic translocation of HMGB1 within injured neurons in the ischemic core was found at 12 h after MCAO, suggesting that HMGB1, a well-recognized damage-associated molecular pattern (DAMP), was released in the infarct core and may drive the formation of ICAM (Fig. 5B). We found that most ICAM-specific marker genes were significantly upregulated in the primary microglia after LPS stimulation, whereas minor changes occurred in the expression of IPAM-specific marker genes (Fig. 5C, D). In order to investigate whether DAMPs released by dying neurons induce ICAM generation, we also prepared neuron-derived DAMPs by repeatedly freeze-thawing HT-22 cells, a mouse neuronal cell line, as previously described [33]. The results showed that primary microglia treated with HT-22-derived DAMPs remarkably upregulated ICAM marker genes with no significant difference in IPAM markers (Additional file 5: Fig S12A, B).

In addition to extracellular microenvironmental cues, we questioned which intracellular regulons drive ICAM generation. Single-cell regulatory network inference and clustering (SCENIC) analysis [16] was used to elucidate key molecular mechanisms underlying the formation of microglial sub-clusters after ischemic stroke. By comprehensively considering the regulon activity score (RAS) and regulon specificity score (RSS), we identified FOSL1 and BACH1 as the most potent ICAM-specific transcription factors (TFs) (Fig. 5E, F). It was suggested that *Fosl1* was upregulated and enriched in ischemic regions after acute ischemia in the ST (Additional file 5: Fig S9J). In addition, enrichment analysis (using Metascape) of the BACH1 downstream target genes found that they were significantly associated with inflammation (e.g., “positive regulation of cell migration,” “positive regulation of MAPK cascade,” “TNF-α NF-κB signaling pathway,” “regulation of cytokine production,” “MyD88-independent TLR4 cascade,” “IL-17 signaling pathway,” “response to oxidative stress,” and “pattern recognition receptor signaling pathway,”), which was consistent with the pro-inflammatory role of ICAM (Additional file 5: Fig S12C). To validate the ICAM-modulating capacity of BACH1, we transfected BV2 cells with lentivirus carrying short hairpin RNA (shRNA) targeting Bach1 (Additional file 1: Fig S12D). Bach1-knockdown in BV2 cells downregulated the expression levels of ICAM-specific marker genes (*Edn1*, *Plau*) upon LPS stimulation (500 ng/mL, 12 h), indicating the potential of BACH1 in transforming homeostatic microglia into ICAM (Additional file 1: Fig S12E). Moreover, classical pro-inflammatory factors, including *Tnf*, *Il1a*, *Il6*, and *Il1b*, were also downregulated in LPS-stimulated Bach1-knockdown cells (Fig. 5G-J).

### Glucocorticoids triggers IPAM formation

**Fig. 6:**
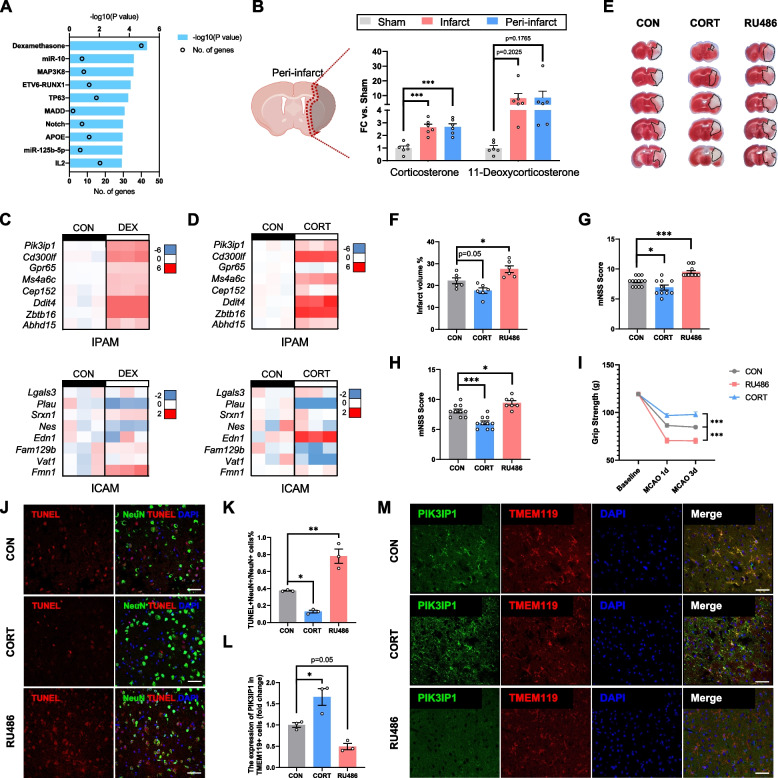
Glucocorticoids triggers IPAM formation. **A** Upstream IPA of top 200 differentially expressed genes in IPAM (Fisher’s exact test, Benjamini–Hochberg FDR). **B** Histograms visualizing targeted metabolomics results in the sham, infarct, and peri-infarct group brain samples from sham and MCAO-12 h mice. *n* = 6/group. **C**–**D** Heatmap showing the expression of several ICAM-specific or IPAM-specific marker genes upon DEX (5 nM, 24 h) (**C**) or CORT (1 μM, 24 h) (**D**) stimulation. *n* = 3/group. **E**–**F** TTC staining of brains from CON, CORT, and RU486 group (**E**). The infarct volume was quantified (**F**). *n* = 6/group. **G**–**H** mNSS were performed at 1 day (**G**) and 3 days (**H**) after MCAO to evaluate the neurological deficits of each group. *n* = 7–14/group. **I** Grip strength was performed at 1 day and 3 days after MCAO to evaluate the neurological deficits of each group. *n* = 7–14/group. **J** Representative TUNEL images co-stained with neuronal-marker NEUN within the ischemic regions in the MCAO 1d brains from CON, CORT, and RU486 groups (scale bar: 50 µm). **K** Proportion of TUNEL^+^/NeuN^+^ cells in NeuN^+^ cells was quantified. *n* = 3/group. **L** Expression of PIK3IP1 in TMEM119.^+^ cells was quantified. The fluorescence intensity of PIK3IP1 in the CORT and RU486 groups was normalized to the mean value of that measured in the CON group. *n* = 3/group. **M** Representative immunostaining of IPAM-specific marker PIK3IP1 and microglia-specific marker TMEM119 in the peri-infarct within MCAO-1d sections from CON, CORT, and RU486 groups (scale bar: 20 µm). In **B**, **F**–**H**, **K**, and **L**, one-way ANOVA with Tukey’s post hoc test. In **I**, two-way ANOVA with Tukey’s post hoc test. Data are presented as mean ± SEM. *P < 0.05, **P < 0.01, ***P < 0.001

To identify the key upstream regulators of IPAM, IPA was performed using the top-200 IPAM marker genes. Strikingly, dexamethasone (DEX) was the strongest stimulating factor for IPAM induction (Fig. 6A). To explore changes in glucocorticoid concentration after stroke, we further exploited high-throughput targeted metabolomics to screen for steroids showing changes following ischemia. According to quantitative data, the levels of corticosterone (mean sham: 39.13 pg/mg, mean ischemic core: 103.53 pg/mg, mean ischemic penumbra: 104.77 pg/mg) and 11-deoxycorticosterone (mean sham: 0.72 pg/mg, mean ischemic core: 5.97 pg/mg, mean ischemic penumbra: 6.23 pg/mg) increased markedly after MCAO in both the ischemic core and peri-infarct area (Fig. 6B). For verification, we used DEX to stimulate primary microglia and strongly induced IPAM generation with significantly enhanced expression of IPAM-specific marker genes (Fig. 6C). Furthermore, corticosterone, the primary adrenal corticosteroid in mice, also significantly upregulated IPAM-specific marker genes expression in primary microglia, indicating the robust capacity of glucocorticoids to drive IPAM generation (Fig. 6D).

To validate these findings in mice, MCAO mice were administered corticosterone or the glucocorticoid receptor antagonist (RU486). Strikingly, MCAO mice that received corticosterone displayed relatively moderate neurological deficits (smaller infarct volume, lower modified neurological severity score [mNSS], and stronger grip strength) (Fig. 6E-I) and decreased neuronal apoptosis (Fig. 6J, K). However, RU486 administration in mice contributed to more severe neurological deficits (larger infarct volume, higher mNSS, and weaker grip strength) (Fig. 6E-I) and exacerbated neuronal death (Fig. 6J, K).

To investigate the alterations of IPAM among different groups experimentally, we employed immunostaining and measured the expression of PIK3IP1 in microglia. Compared with the control group, the mean fluorescence intensity of PIK3IP1 was significantly higher in the corticosterone-treated group and relatively lower in the RU486-administrated group (Fig. 6L, M). Thus, these in vitro and in vivo findings verified the determining role of glucocorticoids in driving IPAM generation and in the neuroprotective properties of IPAM during the acute stage of ischemic stroke.

## Discussion

We first discovered stroke-associated microglia, ICAM and IPAM, using both scRNA-seq and spatial transcriptomics in brain samples from sham and MCAO mice at three different time points. These microglial subclusters are disease-specific and are defined under comprehensive considerations, including the spatiotemporal context, environmental determinants, metabolic profiles, and biological functions. In addition, several markers may provide a glimpse of the potential functions of IPAM and ICAM. Specifically, MS4A6C might regulate the immune function of microglia [34], while GPR65 (TDAG8) was reportedly present in a portion of microglia, and exerts neuroprotective effects by attenuating post-ischemic inflammatory responses [35]. In terms of ICAM, PLAU reportedly regulates inflammation-related signalling and exacerbates deleterious post-ischemia processes [36, 37]. Using ICAM (e.g., LGALS3) and IPAM (e.g., PIK3IP1) markers, ICAM and IPAM localization was confirmed through immunofluorescent staining. Previous studies reported that LGALS3 + microglia were abundant in the core region after ischemia [5], which supports our results. Regional microglial heterogeneity has been increasingly recognized. For example, proliferative region-associated microglia (PAM) were found in developing white matter, and white matter-associated microglia (WAM) were observed in aging white matter [38, 39]. In Alzheimer’s disease, DAM surround amyloid plaques and continuously engulf amyloid-β aggregates [30]. Understanding the regional heterogeneity of microglia and their functions may provide important clues for developing novel therapies for different neurological disorders.

In this study, we found that expression of homeostatic genes of microglia (e.g., *Tmem119*, *P2ry12*, *P2ry13*, *Cx3cr1*, *Hexb*, *Csf1r*) were highly enriched in Cluster 2, which comprised the majority of microglia in the sham mice, consistent with previous studies. However, after ischemic stroke, the transcriptional profile of microglia was markedly altered, and expression of homeostatic genes was significantly downregulated. Furthermore, ICAM and IPAM emerged in the ischemic core and penumbra, respectively, thereby emphasizing their spatial heterogeneity.

Our proposed ICAM and IPAM further negate the M1/M2 paradigm of post-stroke microglia. This dichotomous categorization was established based on the inflammatory states of in vitro cultured microglia [8]. M1-microglia secrete pro-inflammatory cytokines to cause tissue injury, while M2-microglia express anti-inflammatory cytokines to suppress pro-inflammatory responses and promote tissue repair. However, we found that the M2 markers *Mrc1* (encoding CD206) and *Arg1* were predominantly expressed in macrophages rather than in microglia (Fig. 4H). Additionally, although the exaggerated post-stroke pro-inflammatory response was attributed to ICAM, we found that IPAM showed moderate pro-inflammatory responses as compared to homeostatic microglia, and a significant increase of canonical anti-inflammatory cytokines (*Il10, Il4* and *Tgfb1*) was not observed in IPAM (Additional file 5: Fig S11A). Furthermore, the M1/M2 paradigm only focuses on inflammation, neglecting other important biological functions as well as the spatial heterogeneity of microglia.

Owing to the spatial heterogeneity of microglia in the ischemic brain, we speculated that different microenvironmental cues may drive the emergence of ICAM and IPAM. Similar to previous studies, we found that, in the ischemic core, HMGB1, an endogenous TLR4 ligand, translocated from the nucleus to the cytoplasm by 12 h after MCAO, suggesting that it is released from the damaged cells and acts as a DAMP. Moreover, we used LPS and HT-22-derived DAMPs to stimulate in vitro cultured primary microglia and found that several ICAM markers were significantly upregulated. Furthermore, targeted metabolomics and IPA were performed to confirm whether corticosterone might contribute to the formation of IPAM. However, we found that corticosterone was increased in both the infarct core and peri-infarct area, indicating that DAMPs may override the effect of corticosterone in the ischemic core, which induces ICAM formation. However, in the peri-infarct area, since cellular damage was relatively mild, corticosterone could become the determining factor for the microglial state. These findings may open new avenues for therapeutics targeting different stimuli that drive generation of specific microglial sub-clusters. As an identified DAMP, HMGB1 triggers detrimental microglia through the RAGE–NF-κB pathway. Interventions targeting the HMGB1–RAGE axis could modulate the immune microenvironment and provide neuroprotection through microglia-mediated inflammation [40]. Recently, a DAMP-scavenging hydrogel scaffold was invented to promote neuronal regeneration and motor function recovery [41]. Additionally, glucocorticoids (cortisol in primates and corticosterone in rodents) are released upon activation of the hypothalamic–pituitary–adrenal axis in response to acute stress. Glucocorticoids have been implicated in microglial morphology and function. Elevated glucocorticoid levels also contribute to the enhanced ramification of hippocampal microglia, positioning glucocorticoids as a player in the modulation of microglial morphological changes [42]. After ischemia, sustained administration of corticosterone significantly suppresses markers of microglial and astrocyte activity [43]. However, other reports on chronic unpredictable stress have demonstrated that enhanced corticosterone leads to microglial activation in the medial prefrontal cortex, and administration of glucocorticoid receptor antagonists attenuated microglia-mediated neuronal remodeling by preventing microglial engulfment of neuronal elements [44]. In microglial glucocorticoid receptor-depleted mice, the expression of pro-inflammatory genes was increased, while that of homeostatic genes was decreased in microglia under basal microenvironments [45], indicating a complex—and even opposing—role of glucocorticoids under various conditions. Our study provided new insights that different microenvironmental cues in the ischemic core and penumbra probably drive formation of transcriptionally and functionally distinct microglial sub-clusters, revealing a novel perspective on dynamic changes in microglia during ischemia.

Microglia stimulated with different microenvironmental cues also exhibit distinct metabolic characteristics. Reportedly, HMGB1 released from damaged cells could induce a metabolic shift from glucose-dependent aerobic respiration to glycolysis via the suppression of the dimeric form of PKM2 [46]. In metabolic flux assays, LPS significantly enhanced glycolysis, but inhibited OXPHOS in primary microglia [47]. Contrastingly, when stimulated by glucocorticoids, glycolysis is inhibited, while the TCA cycle and OXPHOS flux are promoted, to ensure energy availability. Corticosterone is also perceived as a vital mediator of lipid modulation and amino acid catabolism in response to stress [48, 49]. Metabolic reprogramming has been linked to the regulation of immune cell function in response to local energy challenges; it initiates the phenotype shifts of microglia. Consistent with numerous publications, we found that ICAM, fueled by glycolysis, are highly involved in inflammation-related biological processes (e.g., cytokine–cytokine receptor interaction and chemokine signaling) and immune or infectious diseases (e.g., systemic lupus erythematosus, asthma, and *Staphylococcus aureus* infection). Glycolysis or interruption of the TCA cycle promotes the microglial pro-inflammatory response, whereas an intact TCA cycle and OXPHOS maintain microglial homeostasis and suppress overactive inflammation [50]. Furthermore, peripheral immune cells respond to chemokines and infiltrate the brain following ischemia. We previously discovered that T cells and neutrophils interact with microglia and worsen stroke outcomes, indicating a pivotal role of microglia as a central hub linking the central nervous system with the peripheral immune system in ischemic stroke [10]. We found that ICAM express high levels of various chemokines to form a chemo-attractive milieu in the ischemic core, recruiting infiltrating peripheral immune cells. However, compared to ICAM, several metabolic pathways related to fatty, primary bile, and amino acids; sphingolipids; taurine; and ketone bodies were enriched in IPAM. Degradation of BCAAs, which are known to shape microglial responses to pro-inflammatory signals [51], is elevated in IPAM. Furthermore, taurine was suggested to have neuroprotective effects by preventing microglia-mediated pro-inflammatory responses [52]. Numerous studies have demonstrated that lipid metabolites are closely associated with neuroinflammation. The potential anti-inflammatory effects of sphingolipid signaling in neurodegenerative and neuroinflammatory processes have been increasingly investigated [53]. Bile acids reportedly suppress the neurotoxic phenotype of microglia and ameliorate inflammation [54]. Additionally, β-hydroxybutyrate—a ketone body—may inhibit NLRP3 inflammasome activation and promote microglial ramification, thus exerting anti-inflammatory effects [55, 56]. These differences in metabolism may play an indispensable role in modifying the reactive states of microglia and may contribute to the distinct degrees of pro-inflammatory responses of ICAM and IPAM. We speculate that IPAM may play a neuroprotective role via inflammation-resolving metabolic profiles. Additionally, given that microglia-derived lipid components are essential for remyelination, IPAM might be pivotal for post-stroke remyelination by releasing lipid metabolites and oligodendrotrophic factors, such as GRN.

Furthermore, DAM—widely recognized as a microglial subset related to neurodegeneration—was identified by scRNA-seq. During AD progression, microglia gradually switched from a homeostatic state to DAM, as evidenced by specific markers (e.g., *Csf1*, *Itgax*, *Cd63*, *Ank*, *Axl*, *Igf1*, *Clec7a*, *Tyrobp*, *Ctsb*, *Ctsd*, *Ctsl*, *Cd9*, *Ctsz*, *Ccl6*, *Trem2*, *Spp1*, *Lpl*, *Cst7*, *Apoe*). Furthermore, DAM also showed a unique pattern of spatial distribution, being localized around amyloid-β plaques. DAM activation occured in a sequential two-step process. The first was TREM2-independent, while the second was TREM2-dependent. Moreover, stage 1 DAM upregulated markers related to TREM2 regulators/adaptors (e.g., *Tyrobp*, *Apoe*), while stage 2 DAM were involved in enhanced phagocytic, lysosomal, and lipid metabolism pathways (e.g., *Lpl*, *Cst7*, *Axl*, *Itgax*, *Spp1*, *Cd9*, *Ccl6*, *Csf1*), indicating their capacity to dismantle and digest plaques, and their important role in restricting neurodegeneration. However, the TREM2 pathway was not altered in ICAM and IPAM, suggesting a difference in the formation of these two stroke-associated microglial subclusters and DAM. Although ICAM showed an increase in several DAM-markers, we found that an exaggerated pro-inflammatory response, rather than DAM-like phagocytic ability, was the most significant feature of ICAM, highlighting the adverse effects mediated by ICAM.

We considered how these stroke-associated microglial subclusters were transcriptionally regulated. SCENIC analysis revealed that several key TFs may be involved in the formation of ICAM. We took BACH1 as a representative TF and confirmed that Bach1 knockdown in BV2 cells diminished the ICAM signature and suppressed the pro-inflammatory response. Thus, BACH1 was strongly implicated in most ICAM features, including ICAM phenotype markers and cytokine generation. Reportedly, BACH1 is a mechano-sensor of hemodynamic stress that promotes formation of atherosclerotic plaques. Disturbed blood flow induces the nuclear translocation of BACH1, which then interacts with YAP to promote the transcription of pro-inflammatory genes [57]. In microglia, macrolactins have shown potential in inhibiting LPS-induced pro-inflammatory responses by downregulating BACH1 expression [58]. While TFs are difficult to target pharmacologically, chemical compounds that could downregulate BACH1 may be an alternative for modulating microglial function in the treatment of ischemic stroke.

There are some limitations of our study. First, each sample came from one hemisphere due to the high cost of scRNA-seq. To avoid the limitation of small sample size, we reanalyzed several published microglial datasets, which showed consistency with our results (Additional file 5: Fig. S7). Besides, we also performed RNAscope and immunostaining for verification of IPAM and ICAM. Another limitation of our study is the lack of investigation of whether Bach1 could drive ICAM generation in vivo. The quality of MCAO-3d scRNA-seq sample was also relatively low compared with other samples due to the lower nUMIs and nGenes. Further validation and investigation were still warranted when utilizing our prompt MCAO-3d scRNA-seq results. Additionally, other core TFs identified as being related to ICAM, such as FOSL1, should be further investigated. Moreover, homozygous null mutants for Nr3c1, which encodes a glucocorticoid receptor, was reported to result in perinatal lethality [59], which limits our ability to perform an in vivo study on the capacity of glucocorticoids for triggering IPAM formation. We plan to address these issues by acquiring Bach1 knockout mice and constructing Nr3c1^fl/fl^; Tmem119-CreERT2 mice in the future. Furthermore, the integrated analysis of scRNA-seq and ST was insufficient in our study. The prerequisite and limitations of robust cell type decomposition (RCTD) and multimodal intersection analysis (MIA) made the combined analysis unfeasible, especially the under-estimation of neurons. Newly developed algorithms and methods are needed to optimize the integrated analysis to reveal more discoveries.

## Conclusions

In summary, we identified ICAM and IPAM as novel microglial subclusters associated with ischemic stroke, induced by distinct regional microenvironmental cues. Based on the bioinformatic analysis and further experimental validations, ICAM are supposed to secrete pro-inflammatory cytokines and chemokines, attracting peripheral immune cells to the brain, exaggerating post-stroke neuroinflammation, and exacerbating ischemic damage. However, IPAM probably exhibit neuroprotective effects by producing several inflammation-resolving metabolites and secreting myelinotrophic factors to alleviate ischemic brain injury. Our study highlights the potential of targeting specific microglial subtypes as a therapeutic strategy for treating ischemic stroke, and more efforts are needed for further investigation.

### Supplementary Information

**Additional file 1: Table S1.** Quality control data for scRNA-seq and ST.

**Additional file 2: Table S2. **Canonical marker genes used to identify main cell types. **Table S6**. Representative genes involved in metabolic pathways. **Table S7.** GSVA analysis. **Table S8.** Representative chemokines that recruit peripheral immune cells. **Table S9.** M1/M2 polarization related gene sets. **Table S10.** Primer sequence. 

**Additional file 3: Table S3.** Markers for each cell type in scRNA-seq datasheet.

**Additional file 4: Table S4.** Markers for microglia at different time points in scRNA-seq datasheet.

**Additional file 5: Figure S1. **Identification of microglia. **Figure S2. **Temporal alterations of microglia after ischemic stroke. **Figure S3. **Identification of microglial subclusters. **Figure S4. **Expression level of Cluster 1 markers among four identified microglial subclusters. **Figure S5. **Expression level of Cluster 3 markers among four identified microglial subclusters. **Figure S6. **Expression level of DAM markers among four identified microglial subclusters. **Figure S7. **Identification of Cluster 1 and Cluster 3 in published datasets. **Figure S8. **Identification of ICAM and IPAM. **Figure S9.** Spatial distribution of selected genes and pathways. **Figure S10. **Analysis of ICGs and IPGs. **Figure S11. **Functional analysis of ICAM and IPAM. **Figure S12. **ICAM generation driven by DAMPs and BACH1.

**Additional file 6: Table S5.** Markers for each microglial subcluster in scRNA-seq datasheet.

## Data Availability

The single-cell and spatial sequencing data have been deposited to the Sequence Read Archive (SRA) (National Center for Biotechnology Information) under PRJNA912889 [12] and PRJNA912890 [24]. External datasets used in the present study include data from accession no. GSE174574 [18], GSE197731 [19], and GSE227651 [20]. Source data are provided with this paper.
