# Supplementary material for: Acute ischemia induces spatially and transcriptionally distinct microglial subclusters
Source: Genome Med. 2023 Dec 11;15:109. doi: 10.1186/s13073-023-01257-5 (PMC10712107; doi:10.1186/s13073-023-01257-5)
Supplement: Supplementary file 2 — Additional file 2: Table S2. Canonical marker genes used to identify main cell types. Table S6. Representative genes involved in metabolic pathways. Table S7. GSVA analysis. Table S8. Representative chemokines that recruit peripheral immune cells. Table S9. M1/M2 polarization related gene sets. Table S10. Primer sequence. [file 13073_2023_1257_MOESM2_ESM.docx]

**Supplementary Tables for:**

**Acute ischemia induces spatially and transcriptionally distinct microglial subclusters**

Huiya Li^1,5,#^, Pinyi Liu^1,#^, Bing Zhang^5,#^, Zengqiang Yuan^6,7^, Mengdi Guo^1^, Xinxin Zou^1^, Yi Qian^1^, Shiji Deng^1^, Liwen Zhu^1^, Xiang Cao^1^, Tao Tao^1^, Shengnan Xia^1^, Xinyu Bao^1^, and Yun Xu^1,2,3,4,*^

^1^Department of Neurology, Drum Tower Hospital, Medical School and The State Key Laboratory of Pharmaceutical Biotechnology, Institute of Translational Medicine for Brain Critical Diseases, Nanjing University, Nanjing 210008, China. ^2^Jiangsu Key Laboratory for Molecular Medicine, Medical School of Nanjing University, Nanjing 210008, China. ^3^Jiangsu Provincial Key Discipline of Neurology, Nanjing 210008, China. ^4^Nanjing Neurology Medical Centre, Nanjing 210008, China. ^5^Department of Radiology, The Affiliated Drum Tower Hospital of Nanjing University Medical School, Nanjing 210008, China. ^6^The Brain Science Centre, Beijing Institute of Basic Medical Sciences, Beijing 100850, China. ^7^Centre of Alzheimer’s Disease, Beijing Institute for Brain Disorders, Beijing 100069, China

^#^Huiya Li, Pinyi Liu, and Bing Zhang are authors contributed equally to this work

^*^Correspondence: [xuyun20042001@aliyun.com](mailto:xuyun20042001@aliyun.com) (Y. Xu)

**Table S2**. Canonical marker genes used to identify main cell types

| Cell type | Canonical gene markers |
| --- | --- |
| Neuron | *Syt1, Snap25, Grin1, Thy1* |
| Microglia | *Cd74, Csf1r, C3, Tmem119* |
| Astrocyte | *Agp4, Gfap, Mfge8, Aldh1l1, Cldn10, Gja1, Atp1b2, Slc1a3* |
| OPC | *Pdgfra, Vcan, Cspg4* |
| Oligodendrocyte | *Mbp, Mobp, Plp1, Olig2, Sox10, Cldn11* |
| Macrophage | *Mrc1, Lyve1, Pf4* |
| Neutrophil | *S100a9* |
| Endothelial cell | *Flt1, Cldn5* |

**Table S6**. Representative genes involved in metabolic pathways

| Pathways | Genes |
| --- | --- |
| Glycolysis | *Hk1, Hk3, Pgk1, Pgam1, Eno2, Eno3, Pkm, Idha, Hkdc1, Aldoc, Tpi1, Pklr, Pck1, Fbp2, Adh7* |
| TCA cycle | *Ogdh, Suclg2, Sucla2, Sdha, Fh1, Pck2, Pdha1, Dld, Pck1, Pdha2, Cs, Acly, Aco2, Idh2, Idh3g* |
| Oxidative phosphorylation | *Ndufs2, Ndufs3, Ndufs7, Ndufv1, Ndufa12, Ndufb4, Ndufb10, Sdha, Cytb, Cyc1, Cox17, Cox15, Atp5k, Atp5c1, Atp4b* |
| Sphingolipid metabolism | *Kdsr, Sgpp1, B4galt6, Glb1, Galc, B4galnt1, Sptlc2, Sptlc1, Cers2, Asah1, Smpd1, Smpd2, Sphk2* |
| Biosynthesis of UFAs | *Hacd1, Hacd2, Hacd3, Tecr, Acox3, Acaa1a, Hacd3, Acox1, Hsb17b4, Scp2, Acot2, Elovl2, Elovl3, Elovl4, Elovl5* |
| Fatty acid degradation | *Acat1, Acat2, Acaa1a, Acaa2, Hadh, Echs1, Acox1, Acads, Acadm, Acadsb, Gcdh, Acsl1, Eci1, Eci2* |
| Fatty acid elongation | *Acaa2, Hadhb, Hadh, Echs1, Ppt2, Elovl5, Elovl7, Hacd2, Hacd1, Acot7, Them4, Elovl2, Elovl3, Hsd17b12, Acot2* |

**Table S7**. GSVA analysis

| geneset | tval | pval | Interested group |  |
| --- | --- | --- | --- | --- |
| Valine,-leucine-and-isoleucine-biosynthesis | 46.2809 | 0 | ICAM |  |
| Systemic-lupus-erythematosus | 24.5756 | ####### | ICAM |  |
| Glycine,-serine-and-threonine-metabolism | 23.7514 | ####### | ICAM |  |
|  |  |  |  |  |
| Staphylococcus-aureus-infection | 22.2912 | ####### | ICAM |  |
|  |  |  |  |  |
| Pantothenate-and-CoA-biosynthesis | 19.3757 | 2.23E-81 | ICAM |  |
|  |  |  |  |  |
| Asthma | 11.568 | 1.18E-30 | ICAM |  |
| Arachidonic-acid-metabolism | 10.986 | 7.78E-28 | ICAM |  |
| Rheumatoid-arthritis | 10.911 | 1.76E-27 | ICAM |  |
| Autoimmune-thyroid-disease | 8.07044 | 8.26E-16 | ICAM |  |
| Chemokine-signaling-pathway | 6.30275 | 3.11E-10 | ICAM |  |
| HIF-1-signaling-pathway | 4.72808 | 2.31E-06 | ICAM |  |
| Glycolysis-/-Gluconeogenesis | 3.95489 | 7.74E-05 | ICAM |  |
| Biosynthesis-of-amino-acids | 3.44061 | 0.00058 | ICAM |  |
| Valine,-leucine-and-isoleucine-degradation | 26.8409 | ####### | IPAM |  |
| Taurine-and-hypotaurine-metabolism | 21.6874 | ####### | IPAM |  |
| Synthesis-and-degradation-of-ketone-bodies | 16.5873 | 1.45E-60 | IPAM |  |
| Citrate-cycle-(TCA-cycle) | 16.167 | 1.10E-57 | IPAM |  |
| Sphingolipid-metabolism | 15.8941 | 7.56E-56 | IPAM |  |
| Biosynthesis-of-unsaturated-fatty-acids | 14.5453 | 3.36E-47 | IPAM |  |
| Fatty-acid-degradation | 11.0329 | 4.67E-28 | IPAM |  |
| Fatty-acid-metabolism | 10.433 | 2.76E-25 | IPAM |  |
| Steroid-biosynthesis | 7.09008 | 1.48E-12 | IPAM |  |
| Fatty-acid-elongation | 4.94743 | 7.71E-07 | IPAM |  |
| Ubiquinone-and-other-terpenoid-quinone-biosynthesis | 4.31398 | 1.63E-05 | IPAM |  |
| cAMP-signaling-pathway | 3.89872 | 9.77E-05 | IPAM |  |
| Cortisol-synthesis-and-secretion | 3.75403 | 0.00018 | IPAM |  |
| Butanoate-metabolism | 3.71197 | 0.00021 | IPAM |  |
| Primary-bile-acid-biosynthesis | 3.70127 | 0.00022 | IPAM |  |

**Table S8**. Representative chemokines that recruit peripheral immune cells

| Cell type | Chemokines |
| --- | --- |
| Macrophage | *Ccl3, Ccl4, Ccl5* |
| Neutrophil | *Cxcl1, Cxcl2, Cxcl3, Cxcl5* |
| T cell | *Cxcl9, Cxcl10, Cxcl16, Ccl1, Ccl3, Ccl4, Ccl5, Ccl8, Ccl17,Ccl19, Ccl20, Ccl22, Ccl25, Ccl28, Cx3cl1* |
| NK | *Cxcl9, Cxcl10, Ccl3, Ccl4, Ccl5, Cx3cl1* |

**Table S9**. M1/M2 polarization related gene sets

| polarization | Genes |
| --- | --- |
| M1 polarization | *Il12, Il23, Tnf, Il6, Cd86, Il1a, Il1b, Nos2, Cd64, Cd80, Cd40, Cxcr10, Cxcl9, Cxcl10, Cxcl11, Ccl5, Irf5, Irf1, Ido1, Kynu, Ccr7, Cd16, Cd32, Tlr2, Tlr4* |
| M2 polarization | *Arg1, Arg2, Il10, Cd163, Cd23, Cd200r1, Pdcd1lg2, Cd273, Cd274, Cd206, Cd115, Cd276, Il1rn, Il1r2, Il4r, Lyve1, Vegfa, Vegfb, Vegfc, Vegfd, Egf, Ym-1, Pdl2, Fizz1, Ctsa, Ctsb, Ctsd, Tgfb1, Tgfb2, Tgfb3, Fasl, Irf4, Vtcn1, Msr1, Fn1, Dectin-1* |

**Table S10**. Primer sequence

| Gene | Primer sequence | |
| --- | --- | --- |
| *Gapdh* | forward | GCCAAGGCTGTGGGCAAGGT |
|  | reverse | TCTCCAGGCGGCACGTCAGA |
| *Il1a* | forward | CGAAGACTACAGTTCTGCCATT |
|  | reverse | GACGTTTCAGAGGTTCTCAGAG |
| *Il1b* | forward | GCAACTGTTCCTGAACTCAACT |
|  | reverse | ATCTTTTGGGGTCCGTCAACT |
| *Tnf* | forward | CCCTCACACTCAGATCATCTTCT |
|  | reverse | GCTACGACGTGGGCTACAG |
| *Bach1* | forward | TGAGTGAGAGTGCGGTATTTGC |
|  | reverse | GTCAGTCTGGCCTACGATTCT |
| *Lgals3* | forward | AGACAGCTTTTCGCTTAACGA |
|  | reverse | GGGTAGGCACTAGGAGGAGC |
| *Plau* | forward | GCGCCTTGGTGGTGAAAAAC |
|  | reverse | TTGTAGGACACGCATACACCT |
| *Srxn1* | forward | CCCAGGGTGGCGACTACTA |
|  | reverse | GTGGACCTCACGAGCTTGG |
| *Nes* | forward | CCCTGAAGTCGAGGAGCTG |
|  | reverse | CTGCTGCACCTCTAAGCGA |
| *Edn1* | forward | GCACCGGAGCTGAGAATGG |
|  | reverse | GTGGCAGAAGTAGACACACTC |
| *Fam129b* | forward | ATGGGAGACGTACTGTCCACA |
|  | reverse | TCCTCATAGAAGCGAAGGAACT |
| *Vat1* | forward | CGGTGGCTACGATAAGGTGAA |
|  | reverse | CATGAGGTCGGCGAAGTTGAG |
| *Fmn1* | forward | CAGCAGCCAAACGAACATCC |
|  | reverse | CTCCTGCAACTTTCCCTCCT |
| *Pik3ip1* | forward | ATGCAGCGAATCACCTTGC |
|  | reverse | TTTCATCCACGGTCTCACAGG |
| *Cd300lf* | forward | GGCTCCTTGACAGTGCAGT |
|  | reverse | ATGGGTACTTGGATTGCTGGG |
| *Gpr65* | forward | ATGGCGATGAACAGCATGTG |
|  | reverse | ACGCATAAAGATCCGATGTTGG |
| *Ms4a6c* | forward | TCAAAGTGATAGTGGCAATCCAG |
|  | reverse | CCCTTCTCTGTCTTCCCCCAT |
| *Cep152* | forward | CGGCAGTTTTTAGAGGATCATCG |
|  | reverse | CTGGTCCAAACATGCCTGGAA |
| *Ddit4* | forward | CAAGGCAAGAGCTGCCATAG |
|  | reverse | CCGGTACTTAGCGTCAGGG |
| *Zbtb16* | forward | CTGGGACTTTGTGCGATGTG |
|  | reverse | CGGTGGAAGAGGATCTCAAACA |
| *Abhd15* | forward | CTGGGATCTCAACGACCCAC |
|  | reverse | CCATGCTGGCAAGGGTTCA |
| *Hif1a* | forward | ACCTTCATCGGAAACTCCAAAG |
|  | reverse | CTGTTAGGCTGGGAAAAGTTAGG |
| *Actb* | forward | GGCTGTATTCCCCTCCATCG |
|  | reverse | CCAGTTGGTAACAATGCCATGT |
