# Supplementary material for: Acute ischemia induces spatially and transcriptionally distinct microglial subclusters
Source: Genome Med. 2023 Dec 11;15:109. doi: 10.1186/s13073-023-01257-5 (PMC10712107; doi:10.1186/s13073-023-01257-5)
Supplement: Supplementary file 5 — Additional file 5: Figure S1. Identification of microglia. Figure S2. Temporal alterations of microglia after ischemic stroke. Figure S3. Identification of microglial subclusters. Figure S4. Expression level of Cluster 1 markers among four identified microglial subclusters. Figure S5. Expression level of Cluster 3 markers among four identified microglial subclusters. Figure S6. Expression level of DAM markers among four identified microglial subclusters. Figure S7. Identification of Cluster 1 and Cluster 3 in published datasets. Figure S8. Identification of ICAM and IPAM. Figure S9. Spatial distribution of selected genes and pathways. Figure S10. Analysis of ICGs and IPGs. Figure S11. Functional analysis of ICAM and IPAM. Figure S12. ICAM generation driven by DAMPs and BACH1. [file 13073_2023_1257_MOESM5_ESM.docx]

**Supplementary Figures for:**

**Acute ischemia induces spatially and transcriptionally distinct microglial subclusters**

Huiya Li^1,5,#^, Pinyi Liu^1,#^, Bing Zhang^5,#^, Zengqiang Yuan^6,7^, Mengdi Guo^1^, Xinxin Zou^1^, Yi Qian^1^, Shiji Deng^1^, Liwen Zhu^1^, Xiang Cao^1^, Tao Tao^1^, Shengnan Xia^1^, Xinyu Bao^1^, and Yun Xu^1,2,3,4,*^

^1^Department of Neurology, Drum Tower Hospital, Medical School and The State Key Laboratory of Pharmaceutical Biotechnology, Institute of Translational Medicine for Brain Critical Diseases, Nanjing University, Nanjing 210008, China. ^2^Jiangsu Key Laboratory for Molecular Medicine, Medical School of Nanjing University, Nanjing 210008, China. ^3^Jiangsu Provincial Key Discipline of Neurology, Nanjing 210008, China. ^4^Nanjing Neurology Medical Centre, Nanjing 210008, China. ^5^Department of Radiology, The Affiliated Drum Tower Hospital of Nanjing University Medical School, Nanjing 210008, China. ^6^The Brain Science Centre, Beijing Institute of Basic Medical Sciences, Beijing 100850, China. ^7^Centre of Alzheimer’s Disease, Beijing Institute for Brain Disorders, Beijing 100069, China

^#^Huiya Li, Pinyi Liu, and Bing Zhang are authors contributed equally to this work

^*^Correspondence: [xuyun20042001@aliyun.com](mailto:xuyun20042001@aliyun.com) (Y. Xu)

**Fig S1**


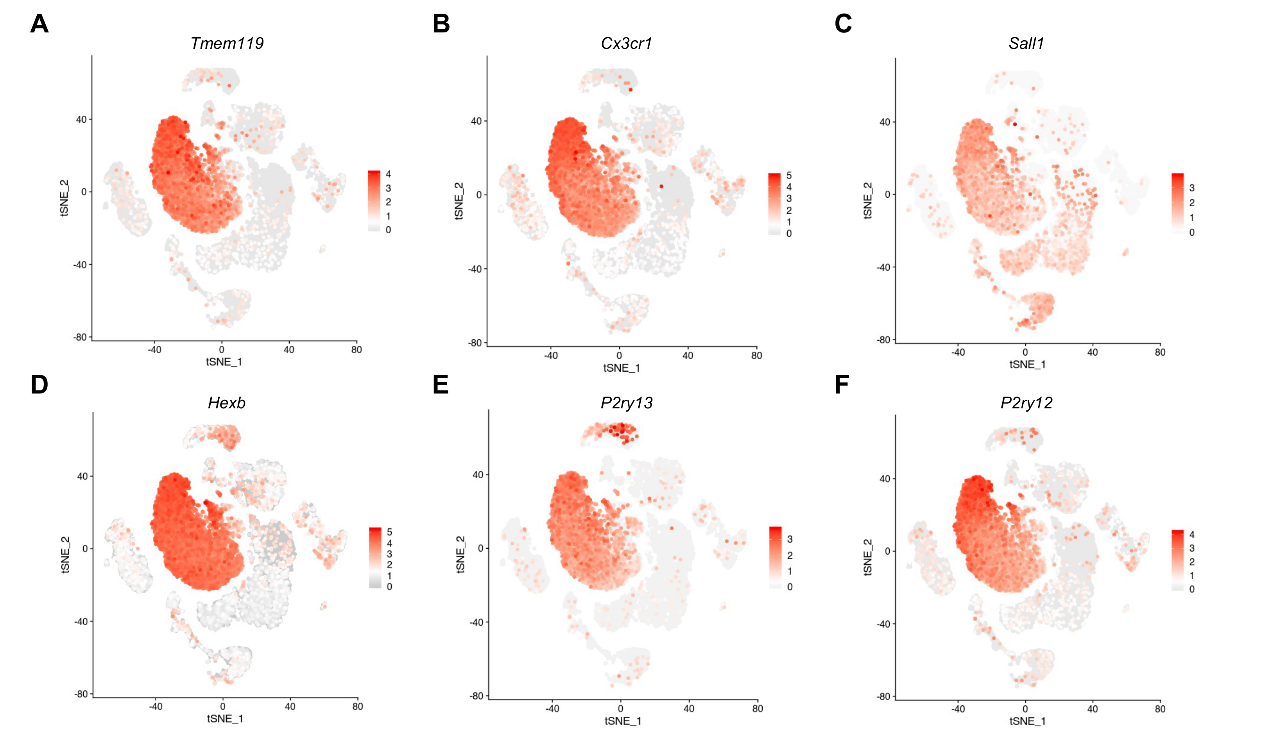


**Figure S1. Identification of microglia.**

Feature plots of several canonical microglia markers including *Tmem119* **(A)**, *Cx3cr1* **(B)**, *Sall1* **(C)**, *Hexb* **(D)**, *P2ry13* **(E)** and *P2ry12* **(F)** in all the cells.

**Fig S2**


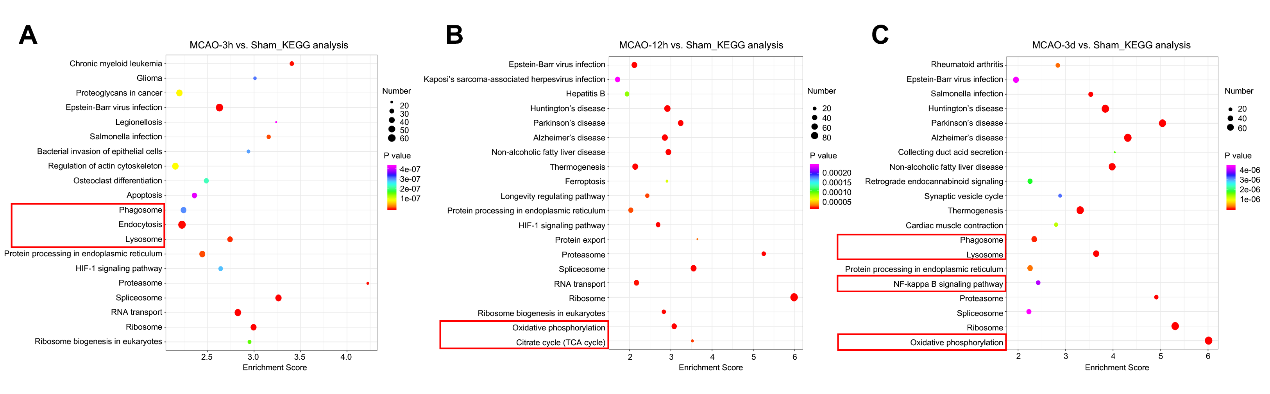


**Figure S2. Temporal alterations of microglia after ischemic stroke.**

KEGG analysis of microglial DEGs at 3-h (A), 12-h (B) and 3-d (C) post cerebral ischemia.

**Fig S3**


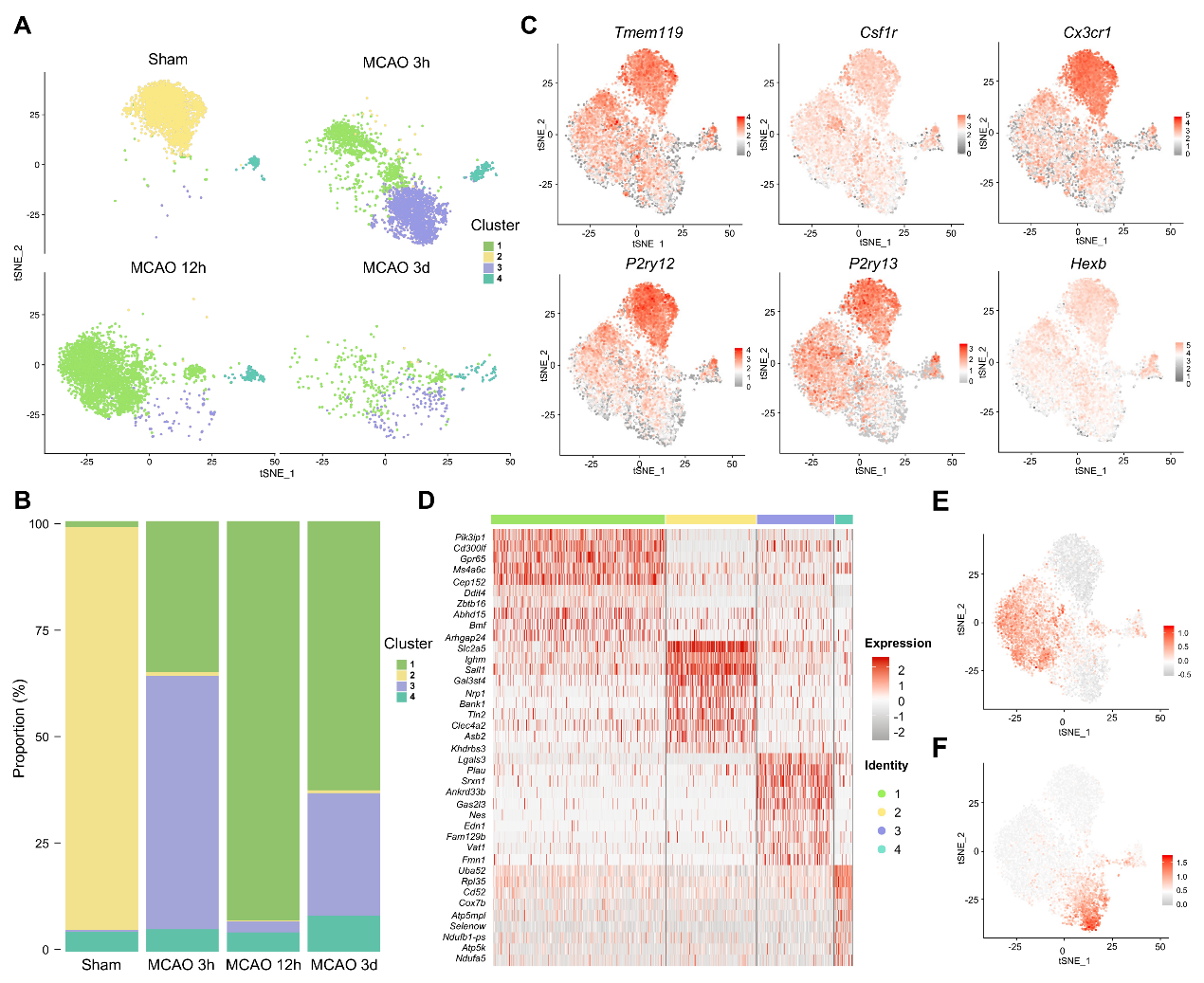


**Figure S3. Identification of microglial clusters.**

**A-B)** t-SNE plot **(A)** and proportional histogram **(B)** depicting the proportion alterations in four microglial sub-clusters in sham and MCAO (3-h, 12-h and 3-d) groups. Cluster annotations and coloring are consistent across panels. **(C)** Feature plots of several homeostatic genes (*Tmem119*, *Csf1r*, *P2ry12*, *P2ry13*, *Cx3cr1*, *Hexb*) in microglia. **(D)** Heatmap showing top-10 marker genes of four distinct microglial sub-clusters. **(E, F)** Feature plots in microglia using top-10 Cluster-1-specific **(E)** or Cluster-3-specific **(F)** marker genes.

**Fig. S4**


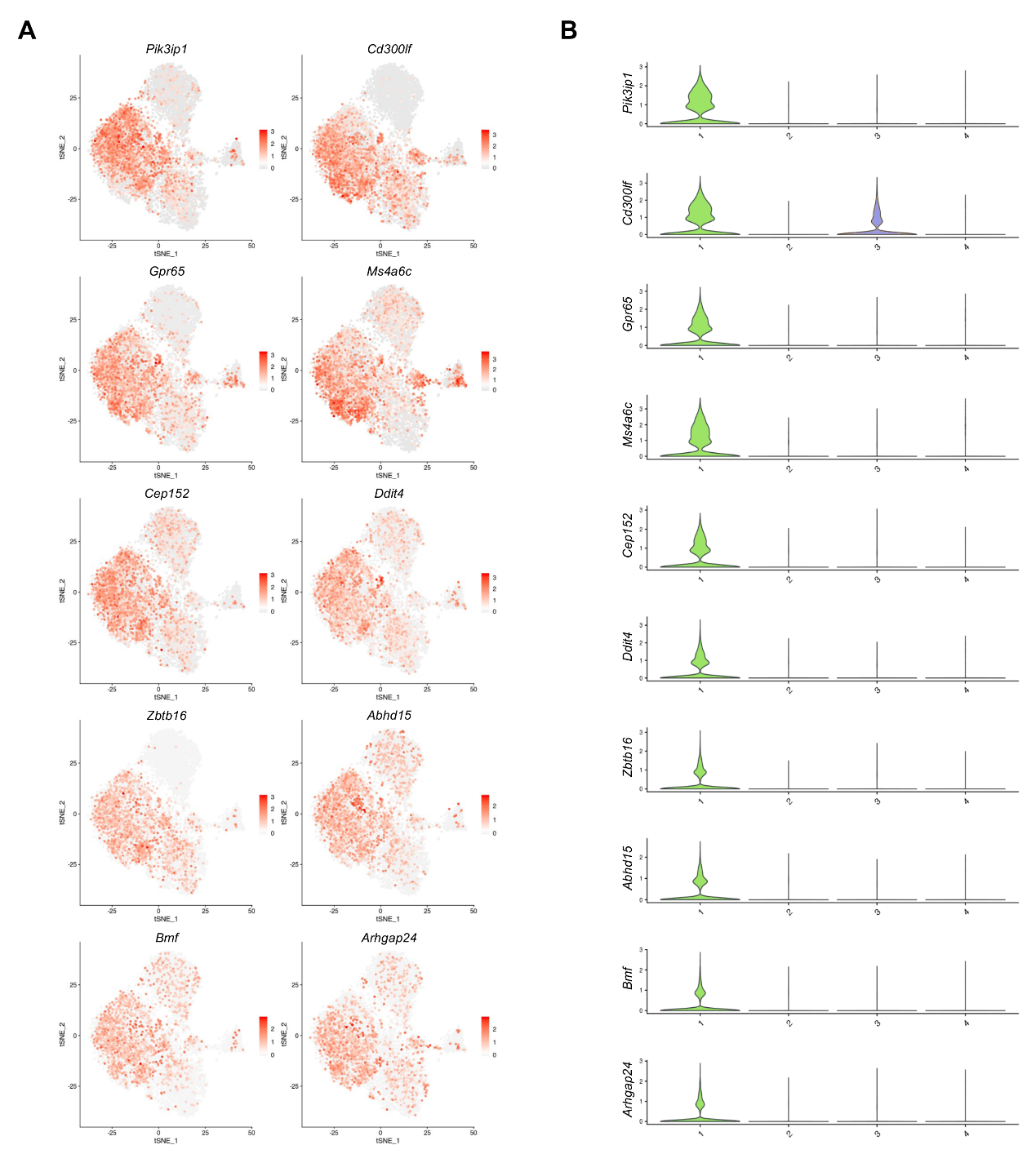


**Figure S4. Expression level of Cluster 1 markers among four identified microglial sub-clusters**.

Feature plots **(A)** and violin plots **(B)** for visualizing the top-10 marker genes of Cluster 1 in microglia.

**Fig. S5**


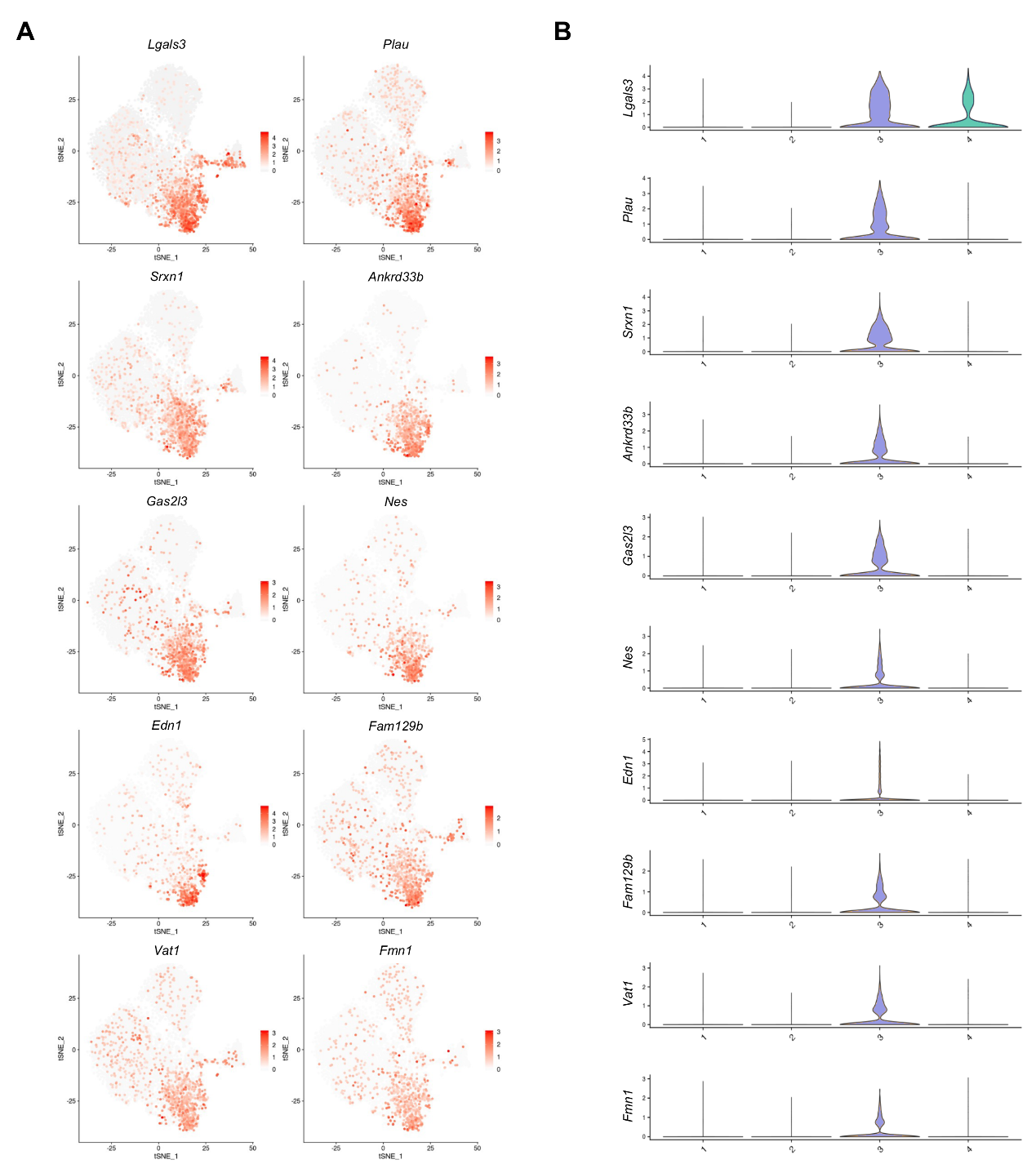


**Figure S5. Expression level of Cluster 3 markers among four identified microglial sub-clusters**.

Feature plots **(A)** and violin plots **(B)** for visualizing top-10 marker genes of Cluster 3 in microglia.

**Fig. S6**


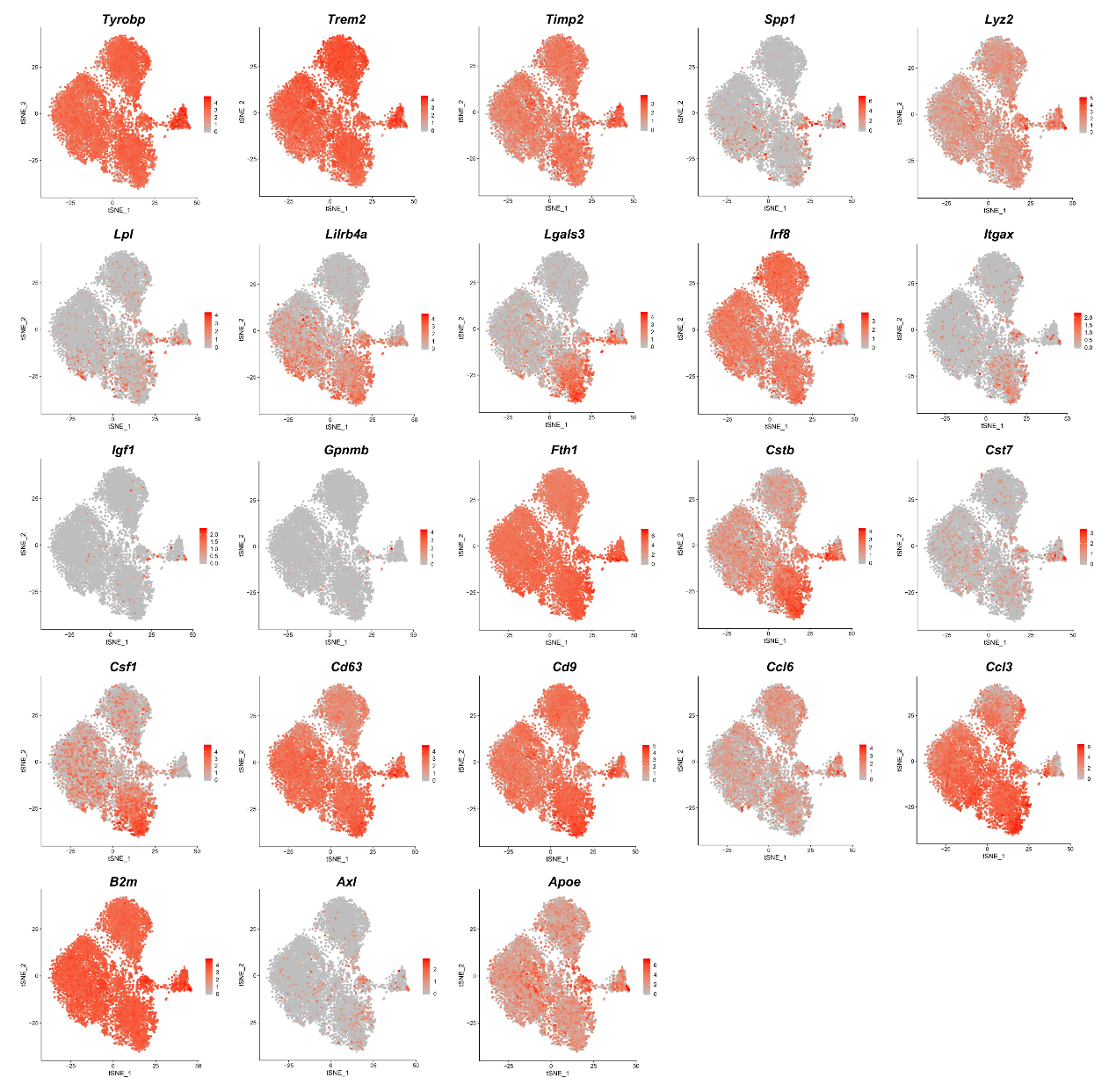


**Figure S6. Expression level of DAM markers among four identified microglial sub-clusters.**

Feature plots visualizing DAM marker genes among four identified microglial sub-clusters.

**Fig. S7**


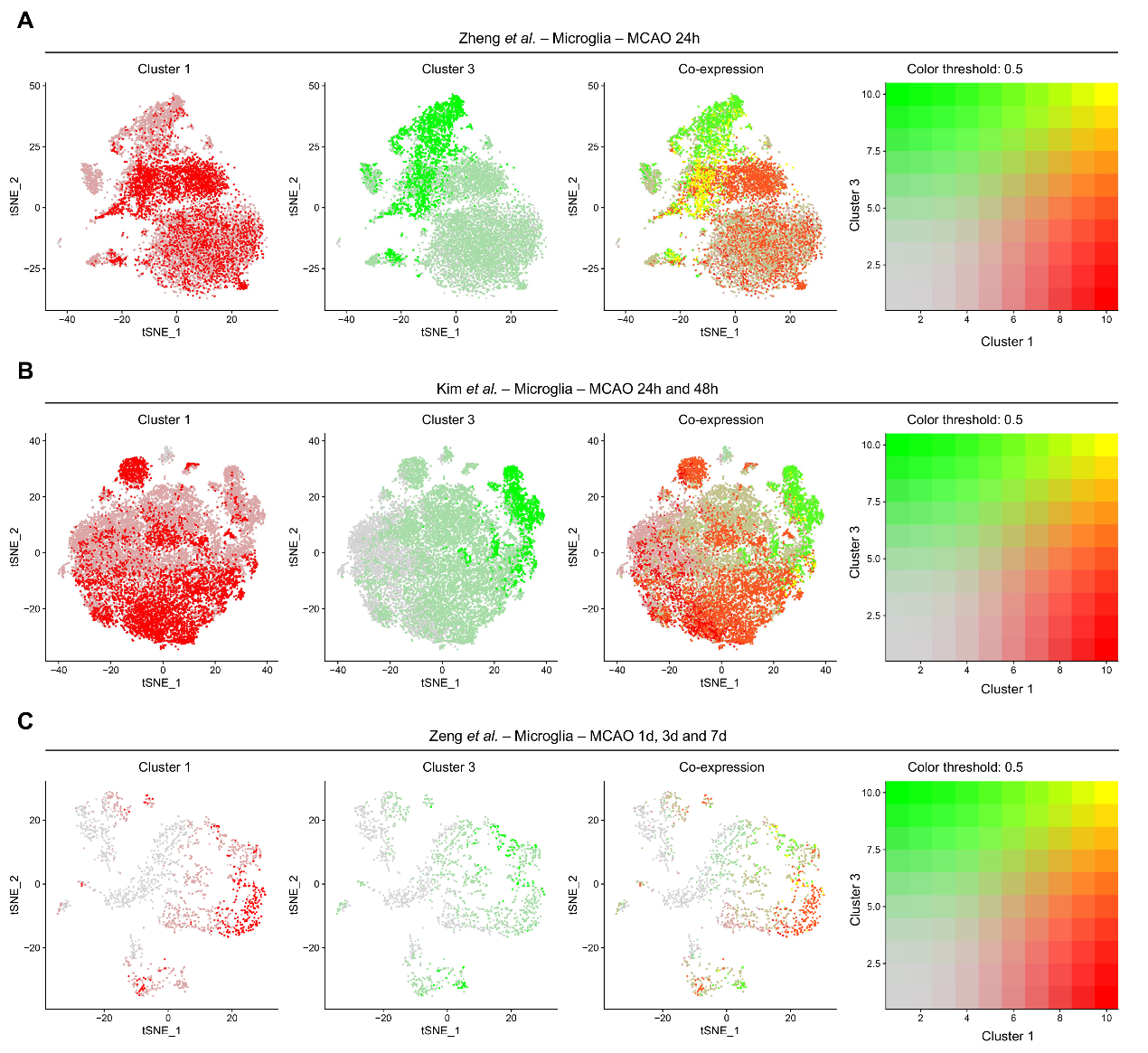


**Figure S7. Identification of Cluster 1 and Cluster 3 in published datasets.**

Reanalysis of published microglial datasets from Zheng *et al.* (MCAO-24h) (A), Kim *et al*. (MCAO-24h and MCAO-48h) (B) and Zeng *et al*. (MCAO-1d, MCAO-3d and MCAO-7d) (C). Cluster 1 (using Cluster 1 gene set from our scRNA-seq) (red), Cluster 3 (using Cluster 3 gene set from our scRNA-seq) (green) were visualized in the t-SNE plot. Co-expression of Cluster 1 marker genes and Cluster 3 marker genes were indicated by yellow.

**Fig. S8**


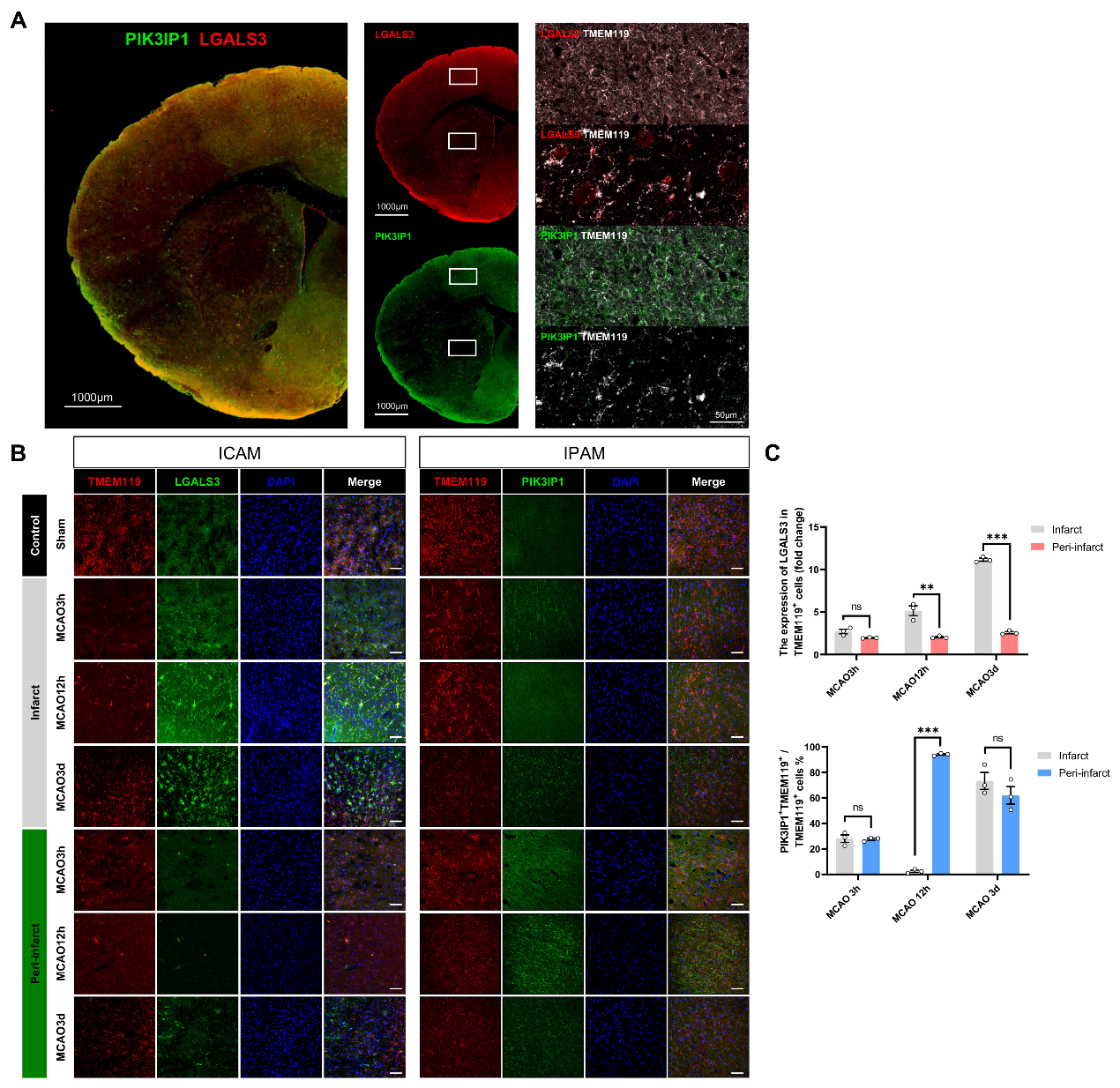


**Figure S8. Identification of ICAM and IPAM.**

**A)** Overview images stained for ICAM-specific LGALS3, IPAM-specific PIK3IP1 and microglial-marker TMEM119 showing the spatial distribution of ICAM and IPAM within sections from MCAO 12-h mice (scale bar: 1000 µm). The right-most images are magnifications of the selected regions (scale bar: 50 µm). **B-C)** Representative immunofluorescence images of ICAM-specific marker LGALS3/IPAM-specific marker PIK3IP1 and microglia-specific marker TMEM119 in the infarct and peri-infarct regions within sections of sham and MCAO (3-h, 12-h, and 3-d post stroke) mice (scale bar: 50 µm) **(B).** Expression of LGALS3 in TMEM119^+^ cells was quantified in the infarct and peri-infarct regions, respectively. The fluorescence intensity of LGALS3 in the MCAO group was normalized to the mean value of that measured in the sham group. The proportion of PIK3IP1^+^TMEM119^+^ cells among TMEM119^+^ cells was manually counted **(C)**. n = 3/group. Data are presented as mean ± SEM. **P < 0.01, ***P < 0.001, by Student’s t-test.

**Fig. S9**


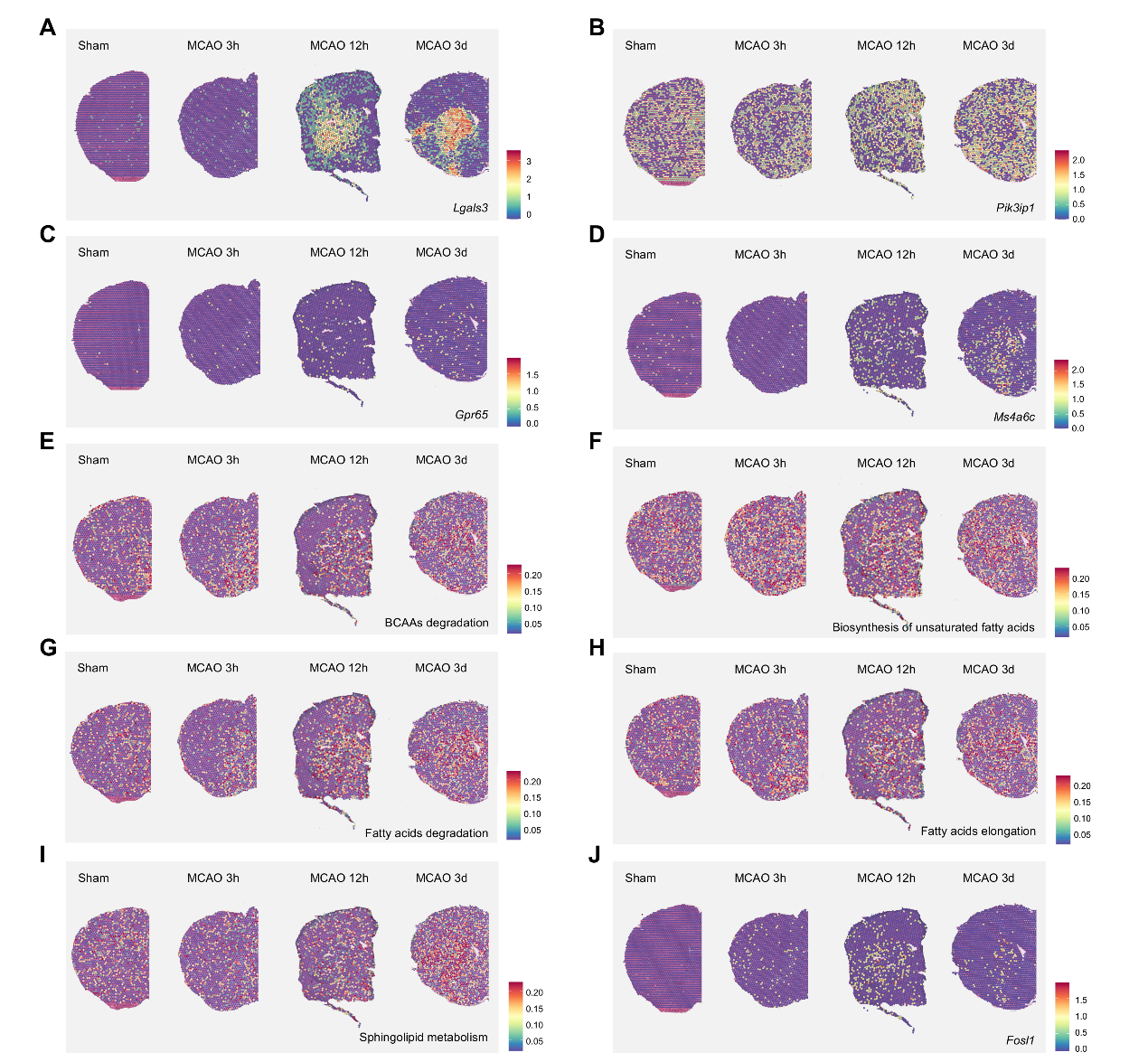


**Figure S9.** **Spatial distribution of selected genes and pathways.**

**A-D)** Feature plots of in selected genes (*Lgals3*, *Pik3ip1*, *Ms4a6c*, *Gpr65*) in the 10×Visium spatial transcriptomics (Sham, MCAO-3h, MCAO-12h, MCAO-3d). **E-I)** 10×Visium spatial transcriptomics highlighting several pathways (“BCAAs degradation,” “Biosynthesis of unsaturated fatty acids,” “Fatty acids degradation,” “Fatty acid elongation,” “Sphingolipid metabolism”) using the AddModuleScore. **J)** Feature plots of in selected transcription factor (*Fosl1*) in the 10×Visium spatial transcriptomics (Sham, MCAO-3h, MCAO-12h, MCAO-3d).

**Fig. S10**


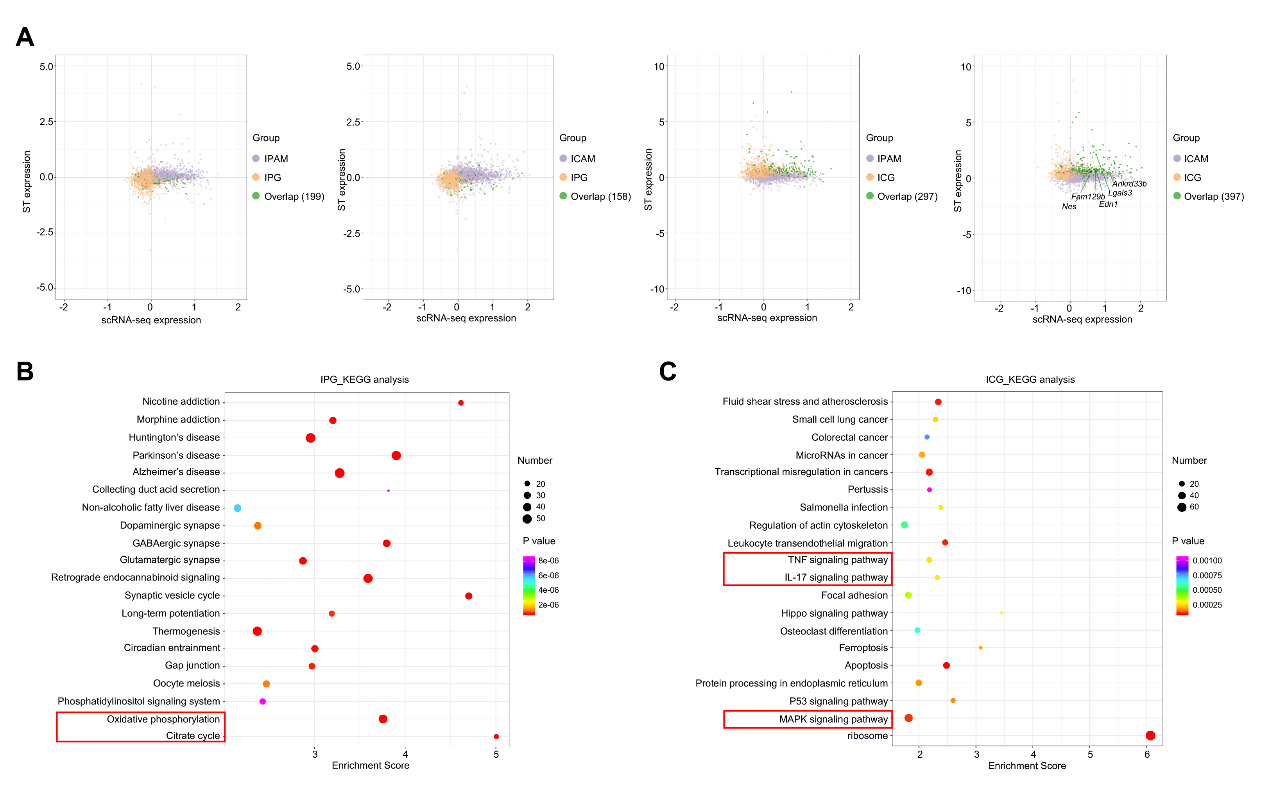


**Figure S10.** **Analysis of ICGs and IPGs**

**A)** Scatter plots showing the overlap between IPAM markers and IPGs, ICAM markers and IPGs, IPAM markers and ICGs, ICAM markers and ICGs respectively. The number of overlapped genes were provided in parentheses, and several ICAM-specific marker genes were highlighted. **B-C)** KEGG analysis of IPGs **(B)** and ICGs **(C)** in the MCAO-12h spatial transcriptomics.

**Fig. S11**


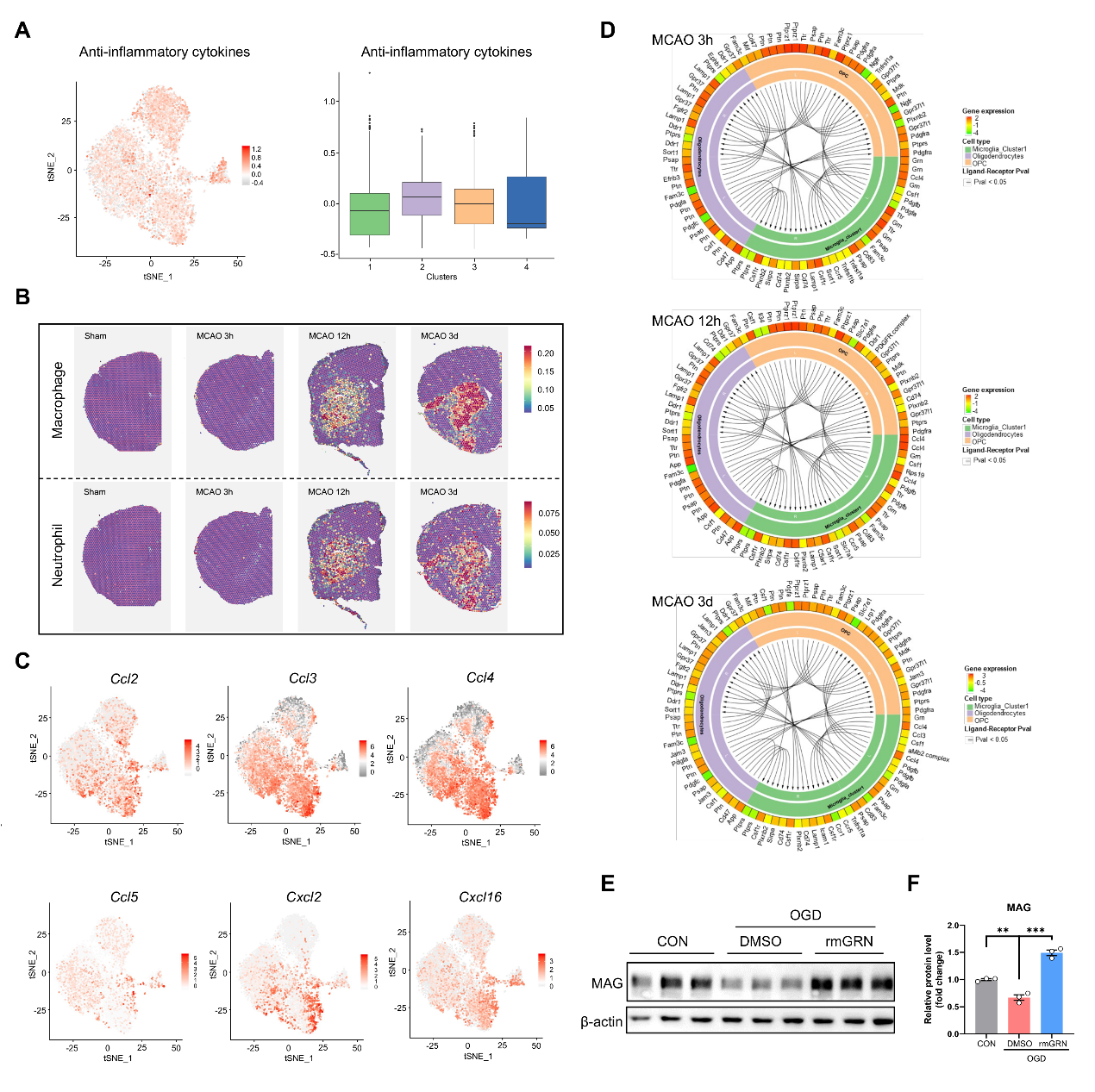


**Figure S11. Functional analysis of ICAM and IPAM.**

**A)** Feature plot (left) and box plot (right) depicting the expression of anti-inflammatory cytokines (*Il4*, *Il10*, *Tgfb1*) in four microglial sub-clusters. **B)** 10×Visium spatial transcriptomics visualizing the expression of macrophage/neutrophil marker genes pertaining to scRNA-seq in sham (control) and MCAO (3-h, 12-h, and 3-d post-ischemic stroke) groups. **C)** Feature plots of selected chemokines (*Ccl2*, *Ccl3*, *Ccl4*, *Ccl5*, *Cxcl2*, *Cxcl16*) in microglia. **D)** Circos plot depicting intercellular communications among IPAM, OPC, and oligodendrocytes at different time-points after stroke using CellPhoneDB. **E-F)** Representative immunoblot bands **(E)** and quantification **(F)** of MAG expression between control (CON group) and OGD group (DMSO group and rmGRN group) in oligodendrocytes. The β-actin-normalized value of MAG in the OGD group was normalized to the mean value of the CON group. n = 3/group.

**Fig. S12**


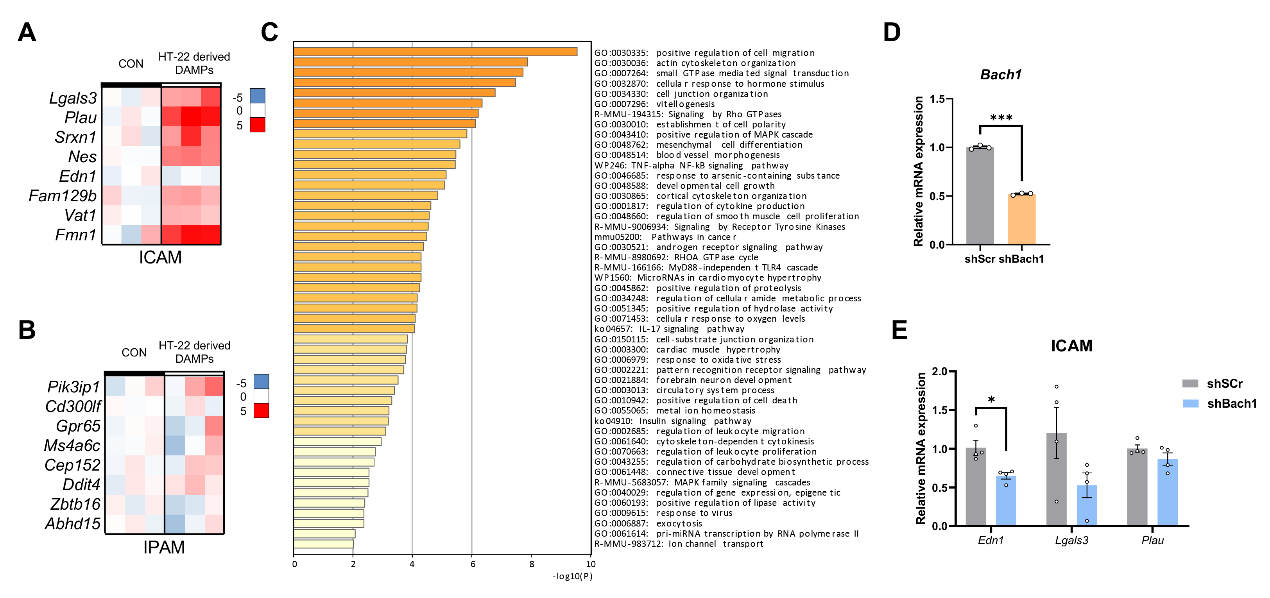


**Figure S12. ICAM generation driven by DAMPs and BACH1.**

**A-B)** Heatmaps showing ICAM-specific **(A)** or IPAM-specific **(B)** marker genes expression upon treatment with necroptotic HT-22-derived DAMPs for 7h. n=3/group. **C)** GO analysis showing the top 50 statistically enriched terms using BACH1 downstream target genes through Metascape (accumulative hypergeometric p-values and enrichment factors were used for filtering). **D)** Real-time PCR analysis evaluating the Bach1-knockdown efficacy in the BV2 cell line. A Bach1-deficient BV2 cell line was established by the shRNA approach. n = 3 biologically independent samples. Data are presented as mean ± SEM. ***P < 0.001, by Student’s t-test. **E)** Real-time PCR analysis of the expression of several ICAM-specific marker genes (*Edn1*, *Lgals3*, and *Plau*) in shScr/shBach1 cell line upon LPS stimulation (500 ng/mL, 12h). n = 4 biological independent samples. Data are presented as mean ± SEM. *P < 0.05, by Student’s t-test.
